# Supplementary material for: Cell-specific mechanisms drive connectivity across the time course of Huntington’s disease
Source: Nat Commun. 2025 Jul 1;16:5519. doi: 10.1038/s41467-025-60556-0 (PMC12218932; doi:10.1038/s41467-025-60556-0)
Supplement: Supplementary file 1 — Supplementary Information [file 41467_2025_60556_MOESM1_ESM.pdf]

Supplementary data

Figures

Figure S1 Nodal strength before and after ComBat harmonisation 3
Figure S2 Cohen's *d* across ROIs representing the differences in nodal strength between mHD and controls applying different parcellation resolutions.................................... 4
Figure S3 MIND brain subnetwork showing connections with significant (P < 0.05 FWE-corrected) increases in connectivity in mHD relative to controls 5
Figure S4 Scatterplot depicting the correlations in node strength/connection strength between cohorts 6

Tables

Table S1 Demographic characteristics of study participants 7
Table S2 P values for the differences before and after ComBat harmonization 8
Table S3 Network-based statistics analysis, significant connections (early preHD > controls) [P_{FWE}=0.029].. 10
Table S4 Network-based statistics analysis, significant connections (mHD < controls) [P_{FWE}<0.0006] 11
Table S5 Network-based statistics analysis, significant connections (mHD > controls) [P_{FWE}=0.022] 24
Table S6 Network-based statistics analysis, significant correlations between connections and NfL in late preHD [P_{FWE}=0.044].. 25
Table S7 Network-based statistics analysis, significant correlations between connections and NfL in mHD [P_{FWE}<0.0001] 26
Table S8 Permutation analysis of nodal strength in early preHD 28
Table S9 Permutation analysis of nodal strength in late preHD 30
Table S10 Permutation analysis of nodal strength in mHD 32
Table S11 Correlation between plasma NfL and nodal strength................................. 34
Table S12 Correlations in node strength between cohorts 37
Table S13 Correlations in connection strength between cohorts 38
Table S14 Correlations between organisational principles and nodal strength 39
Table S15 Receptome gradient correlations 40
Table S16 Dominance analysis between nodal strength and PET neurotransmitter distribution 41
Table S17 Dominance analysis between nodal strength in early preHD and autoradiography neurotransmitter distribution.. 42
Table S18 Dominance analysis between nodal strength in late preHD and autoradiography neurotransmitter distribution.. 43
Table S19 Dominance analysis between nodal strength in mHD and autoradiography neurotransmitter distribution 44
Table S20 Demographics from late preHD participants from TrackHD and TrackOn HD 45
Table S21 Methodological details for each PET tracer 46
Table S22 P values in the epicenter analysis... 64
Table S23 P values in the organizational principles analysis 65
Table S24 P values in the receptome analysis 66
Table S25 P values PET neurotransmitter analysis 67

Table S26 P values in the autoradiography neurotransmitter analysis 68

<u>Supplementary note</u>

Supplementary note 69

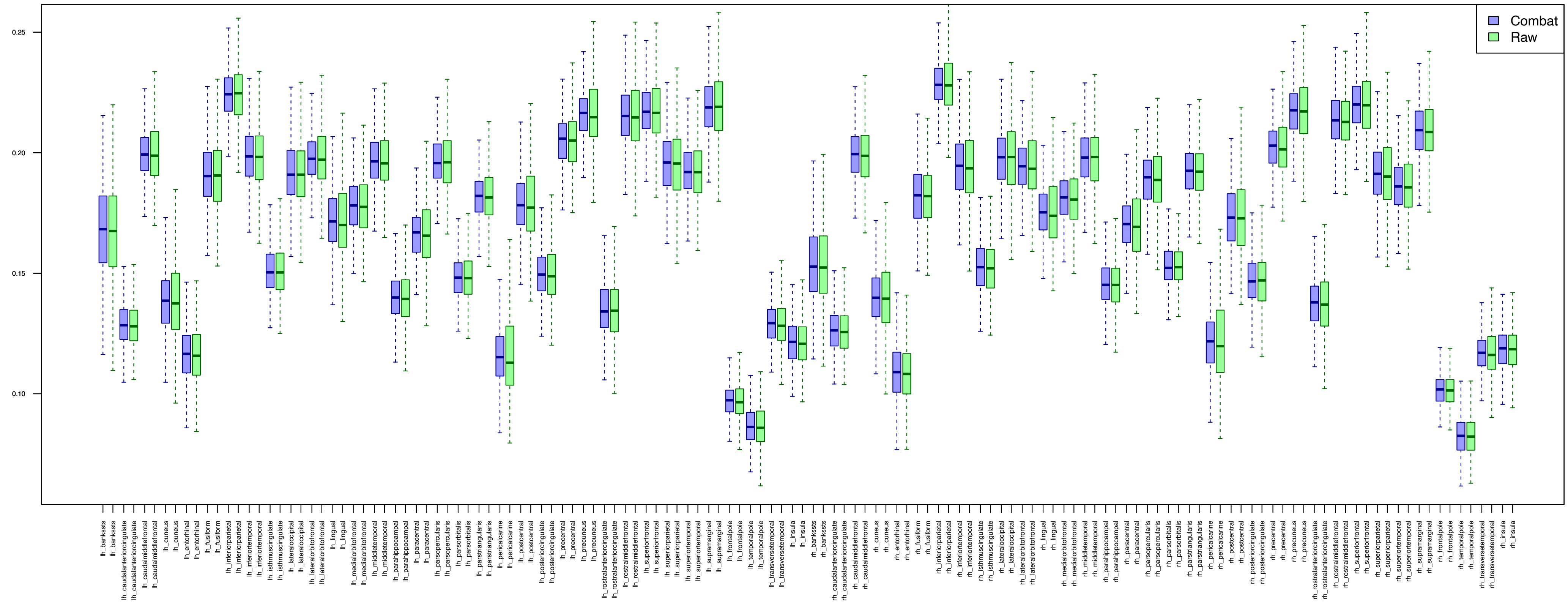

Figure S1 Nodal strength before and after ComBat harmonisation

Box plots representing the distribution of nodal strengths in each ROI before (Blue) and after (Green) performing ComBat harmonisation in mHD participants from TrackHD
mHD, manifest Huntington's disease

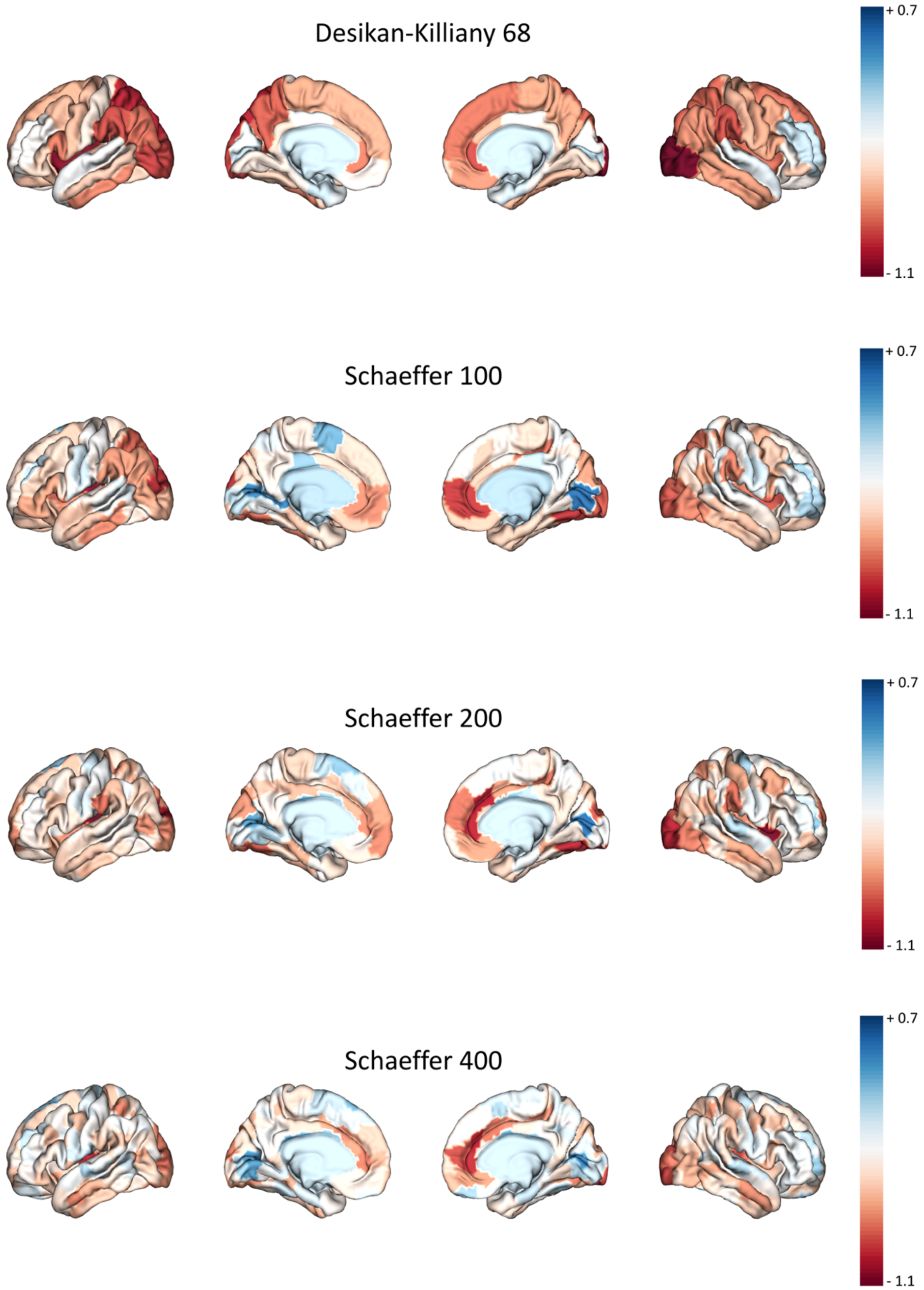

Figure S2 Cohen's *d* across ROIs representing the differences in nodal strength between mHD and controls applying different parcellation resolutions.

mHD, manifest Huntington's disease; ROI, Region-of-interest.

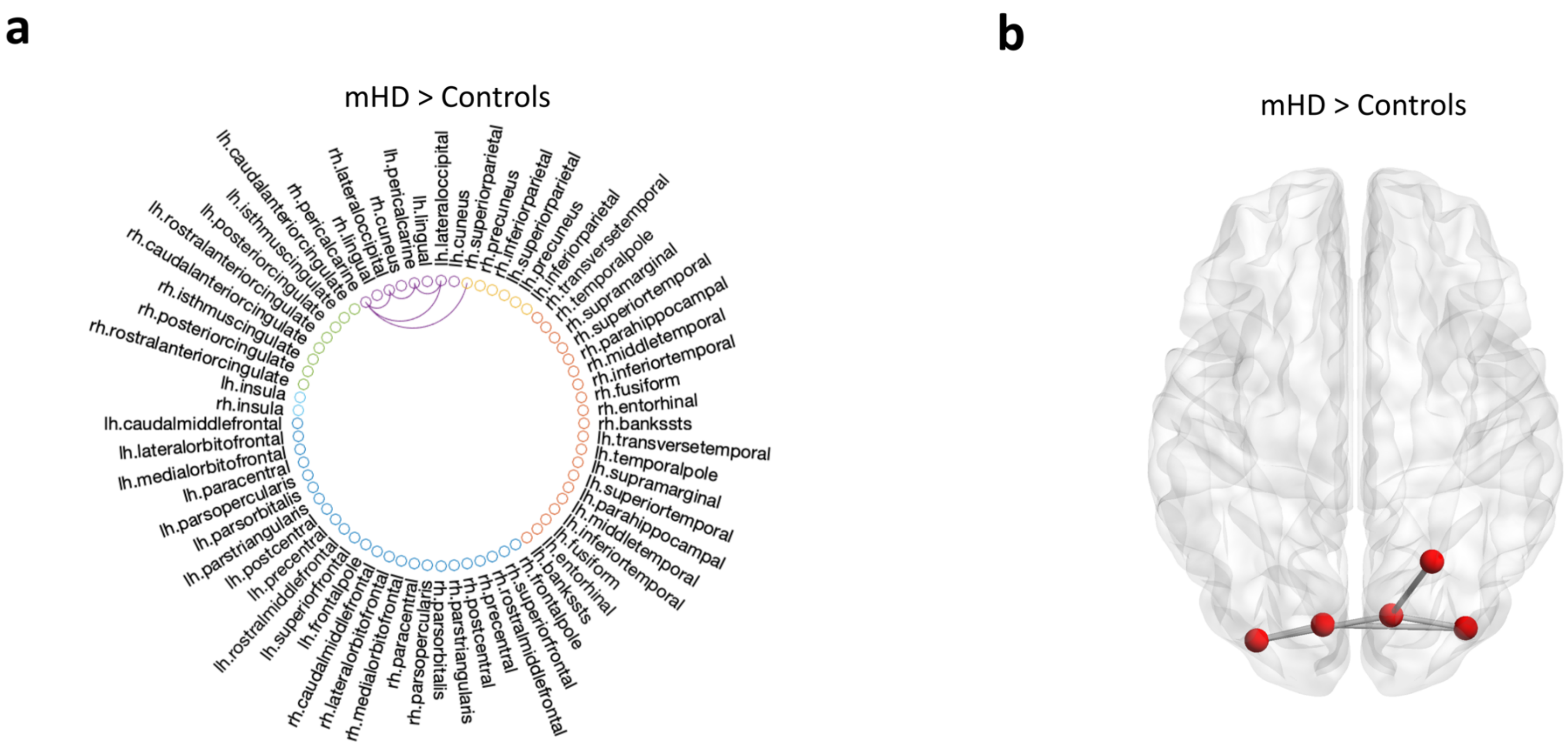

Figure S3 MIND brain subnetwork showing connections with significant (P < 0.05 FWE-corrected) increases in connectivity in mHD relative to controls

Analysis performed using network-based statistics, non-parametric permutation two-tailed t-tests FDR correction at a P<0.05). **a,** Circular graph depicting significant subnetworks. **b,** Axial view. Colour scheme for brain figures. Spheres indicate brain regions; lines indicate connections that are significantly stronger in controls relative to pwHD
FWE, family-wise error rate; MIND, Morphometric Inverse Divergence; mHD, manifest Huntington's disease; pwHD, People with Huntington's disease.

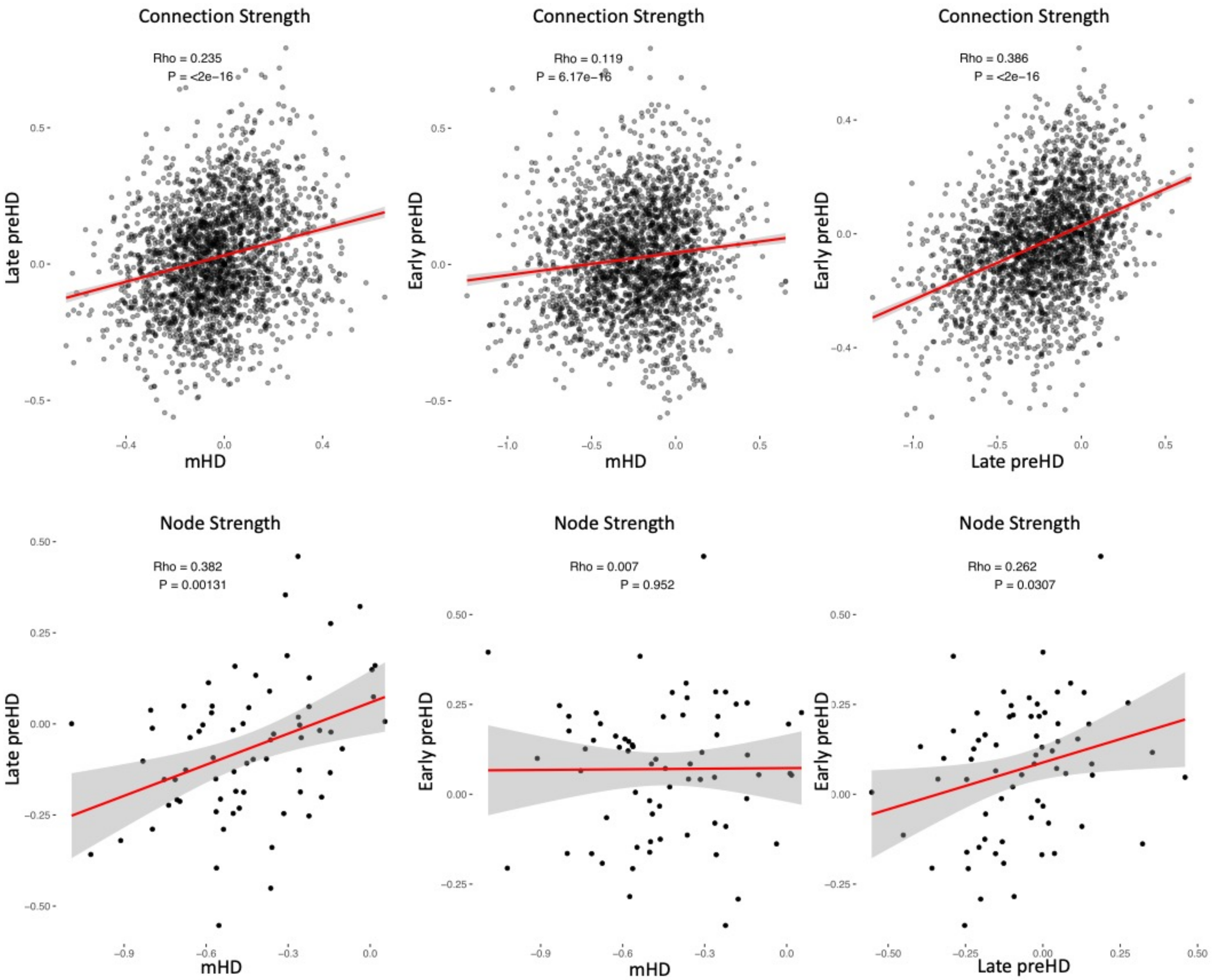

Figure S4 Scatterplot depicting the correlations in node strength/connection strength between cohorts

Pearson correlation test, two-tailed, DF = 4,622 (edge) and 66 (node).
DF, degrees of freedom; mHD, manifest Huntington's disease; preHD, premanifest Huntington's disease

Table S1 Demographic characteristics of study participants

Cohort	Track-HD		TrackOn-HD		HD-YAS	
Group	mHD	Controls	Late preHD	Controls	Early preHD	Controls
N	110	111	85	89	57	60
Age	48.46 (10.05)	46.18 (0.13)	43.66 (8.92)*	48.98 (10.27)*	29.85 (5.66)	29.59 (5.49)
Sex (M:F)	50:61	59:28	39:46	32:57	20:37	30:30
Site	Leiden: 26 London: 29 Paris: 25 Vancouver: 24	Leiden: 28 London: 28 Paris: 24 Vancouver: 30	Leiden: 18 London: 20 Paris: 25 Vancouver: 22	Leiden:25 London:23 Paris: 23 Vancouver : 18	London: 57	London: 60
DBS	377.73 (71.57)	NA	304.31 (55.29)	NA	190.57 (38.08)	NA
CAG	43.791 (3.01)	NA	42.84 (2.33)	NA	42.16 (1.58)	NA
TIV	1461.04 (180.11)	1481.00 (200.58)	1495.45 (187.34)	1465.32 (179.73)	1507.22 (213.78)	1480.65 (204.59)

Group comparisons between patients and controls within each cohort were made using t tests (age, DBS) and chi square tests (sex, stage). *Significant differences ($P<0.05$).
DBS, disease burden score; F, female; M, male TIV, total intracranial volume.

Table S2 P values for the differences before and after ComBat harmonization

ROI	Uncorrected_p_value	FDR-corrected_p_value
lh_bankssts_thickness	0.795434844	0.999839444
lh_caudalanteriorcingulate_thickness	0.859142994	0.999839444
lh_caudalmiddlefrontal_thickness	0.885184212	0.999839444
lh_cuneus_thickness	0.741438658	0.999839444
lh_entorhinal_thickness	0.492126319	0.999839444
lh_fusiform_thickness	0.959643871	0.999839444
lh_inferiorparietal_thickness	0.824496243	0.999839444
lh_inferiortemporal_thickness	0.90055458	0.999839444
lh_isthmuscingulate_thickness	0.637929121	0.999839444
lh_lateraloccipital_thickness	0.852235333	0.999839444
lh_lateralorbitofrontal_thickness	0.754201773	0.999839444
lh_lingual_thickness	0.663215004	0.999839444
lh_medialorbitofrontal_thickness	0.982989636	0.999839444
lh_middletemporal_thickness	0.737773662	0.999839444
lh_parahippocampal_thickness	0.976422925	0.999839444
lh_paracentral_thickness	0.588493363	0.999839444
lh_parsopercularis_thickness	0.868272322	0.999839444
lh_parsorbitalis_thickness	0.894614859	0.999839444
lh_parstriangularis_thickness	0.882399904	0.999839444
lh_pericalcarine_thickness	0.440265648	0.999839444
lh_postcentral_thickness	0.672074846	0.999839444
lh_posteriorcingulate_thickness	0.802653735	0.999839444
lh_precentral_thickness	0.994164203	0.999839444
lh_precuneus_thickness	0.552594648	0.999839444
lh_rostralanteriorcingulate_thickness	0.890740948	0.999839444
lh_rostralmiddlefrontal_thickness	0.913528981	0.999839444
lh_superiorfrontal_thickness	0.832085728	0.999839444
lh_superiorparietal_thickness	0.976587036	0.999839444
lh_superiortemporal_thickness	0.800240867	0.999839444
lh_supramarginal_thickness	0.81671482	0.999839444
lh_frontalpole_thickness	0.585094139	0.999839444
lh_temporalpole_thickness	0.673243996	0.999839444
lh_transversetemporal_thickness	0.872830338	0.999839444
lh_insula_thickness	0.573975544	0.999839444
rh_bankssts_thickness	0.680392556	0.999839444
rh_caudalanteriorcingulate_thickness	0.970439699	0.999839444
rh_caudalmiddlefrontal_thickness	0.880895247	0.999839444
rh_cuneus_thickness	0.741733427	0.999839444
rh_entorhinal_thickness	0.406740536	0.999839444
rh_fusiform_thickness	0.614361959	0.999839444
rh_inferiorparietal_thickness	0.78962457	0.999839444
rh_inferiortemporal_thickness	0.892356439	0.999839444

rh_isthmuscingulate_thickness	0.553808529	0.999839444
rh_lateraloccipital_thickness	0.964485226	0.999839444
rh_lateralorbitofrontal_thickness	0.766310134	0.999839444
rh_lingual_thickness	0.565008632	0.999839444
rh_medialorbitofrontal_thickness	0.924776303	0.999839444
rh_middletemporal_thickness	0.872126885	0.999839444
rh_parahippocampal_thickness	0.868455015	0.999839444
rh_paracentral_thickness	0.59509566	0.999839444
rh_parsopercularis_thickness	0.999839444	0.999839444
rh_parsorbitalis_thickness	0.781325106	0.999839444
rh_parstriangularis_thickness	0.737083424	0.999839444
rh_pericalcarine_thickness	0.543290329	0.999839444
rh_postcentral_thickness	0.737260744	0.999839444
rh_posteriorcingulate_thickness	0.845814845	0.999839444
rh_precentral_thickness	0.884531691	0.999839444
rh_precuneus_thickness	0.620374434	0.999839444
rh_rostralanteriorcingulate_thickness	0.932043287	0.999839444
rh_rostralmiddlefrontal_thickness	0.379632234	0.999839444
rh_superiorfrontal_thickness	0.703215079	0.999839444
rh_superiorparietal_thickness	0.854514258	0.999839444
rh_superiortemporal_thickness	0.855340175	0.999839444
rh_supramarginal_thickness	0.912478146	0.999839444
rh_frontalpole_thickness	0.558192268	0.999839444
rh_temporalpole_thickness	0.535716843	0.999839444
rh_transversetemporal_thickness	0.82312989	0.999839444
rh_insula_thickness	0.539818478	0.999839444

Two-sample T-test, FDR correction at a $P<0.05$

FDR, false-discovery rate; lh, left hemisphere; rh, right hemisphere.

Table S3 Network-based statistics analysis, significant connections (early preHD > controls) [P_{FWE}=0.029]

Connection 1	Connection 2	Test statistic
ctx-lh-lingual	ctx-lh-paracentral	3.76
ctx-lh-lingual	ctx-lh-parsopercularis	3.8
ctx-lh-lingual	ctx-lh-superiorfrontal	3.63
ctx-lh-lingual	ctx-lh-superiortemporal	3.43
ctx-lh-lingual	ctx-rh-inferiorparietal	3.63
ctx-lh-superiorfrontal	ctx-rh-lateraloccipital	3.42
ctx-lh-lingual	ctx-rh-lateralorbitofrontal	3.67
ctx-lh-lingual	ctx-rh-parsopercularis	4.19
ctx-rh-lateraloccipital	ctx-rh-superiorfrontal	3.39

Network-based statistics, non-parametric permutation two-tailed t-tests FDR correction at a $P<0.05$
FDR, false discovery rate; ctx, cortex; lh, left hemisphere; preHD, premanifest Huntington's disease; rh, right hemisphere.

Table S4 Network-based statistics analysis, significant connections (mHD < controls) [$P_{FWE}<0.0006$]

Connection 1	Connection 2	Test statistic
ctx-lh-caudalanteriorcingulate	ctx-lh-cuneus	4.12
ctx-lh-caudalanteriorcingulate	ctx-lh-inferiorparietal	3.47
ctx-lh-caudalmiddlefrontal	ctx-lh-inferiortemporal	3.47
ctx-lh-inferiorparietal	ctx-lh-inferiortemporal	3.93
ctx-lh-cuneus	ctx-lh-isthmuscingulate	3.4
ctx-lh-caudalanteriorcingulate	ctx-lh-lateraloccipital	4.58
ctx-lh-caudalmiddlefrontal	ctx-lh-lateraloccipital	4.17
ctx-lh-fusiform	ctx-lh-lateraloccipital	3.44
ctx-lh-inferiorparietal	ctx-lh-lateraloccipital	4.49
ctx-lh-inferiortemporal	ctx-lh-lateraloccipital	5.8
ctx-lh-lateraloccipital	ctx-lh-lateralorbitofrontal	4.59
ctx-lh-caudalanteriorcingulate	ctx-lh-lingual	3.95
ctx-lh-cuneus	ctx-lh-medialorbitofrontal	3.55
ctx-lh-lateraloccipital	ctx-lh-medialorbitofrontal	6.25
ctx-lh-lingual	ctx-lh-medialorbitofrontal	3.37
ctx-lh-lateraloccipital	ctx-lh-middletemporal	4.1
ctx-lh-lateraloccipital	ctx-lh-parahippocampal	3.41
ctx-lh-caudalanteriorcingulate	ctx-lh-paracentral	4.13
ctx-lh-fusiform	ctx-lh-paracentral	5.32
ctx-lh-inferiortemporal	ctx-lh-paracentral	6.28
ctx-lh-isthmuscingulate	ctx-lh-paracentral	3.6
ctx-lh-lateraloccipital	ctx-lh-paracentral	3.14
ctx-lh-lateralorbitofrontal	ctx-lh-paracentral	5
ctx-lh-medialorbitofrontal	ctx-lh-paracentral	4.07
ctx-lh-middletemporal	ctx-lh-paracentral	4.19
ctx-lh-parahippocampal	ctx-lh-paracentral	3.84
ctx-lh-inferiortemporal	ctx-lh-parsopercularis	3.17
ctx-lh-lateraloccipital	ctx-lh-parsopercularis	5.16
ctx-lh-lateraloccipital	ctx-lh-parsorbitalis	3.33
ctx-lh-lateraloccipital	ctx-lh-parstriangularis	4.79
ctx-lh-caudalanteriorcingulate	ctx-lh-postcentral	4.26
ctx-lh-inferiortemporal	ctx-lh-postcentral	4
ctx-lh-medialorbitofrontal	ctx-lh-postcentral	4.58
ctx-lh-cuneus	ctx-lh-posteriorcingulate	3.67
ctx-lh-lateraloccipital	ctx-lh-posteriorcingulate	3.33
ctx-lh-lingual	ctx-lh-posteriorcingulate	4.4
ctx-lh-postcentral	ctx-lh-posteriorcingulate	3.23
ctx-lh-fusiform	ctx-lh-precentral	3.95
ctx-lh-inferiortemporal	ctx-lh-precentral	5.61
ctx-lh-lateraloccipital	ctx-lh-precentral	3.86
ctx-lh-lateralorbitofrontal	ctx-lh-precentral	4.13
ctx-lh-middletemporal	ctx-lh-precentral	3.59

ctx-lh-caudalmiddlefrontal	ctx-lh-precuneus	3.75
ctx-lh-inferiortemporal	ctx-lh-precuneus	4.31
ctx-lh-lateralorbitofrontal	ctx-lh-precuneus	3.34
ctx-lh-middletemporal	ctx-lh-precuneus	3.11
ctx-lh-parsopercularis	ctx-lh-precuneus	3.6
ctx-lh-bankssts	ctx-lh-rostralanteriorcingulate	3.45
ctx-lh-caudalmiddlefrontal	ctx-lh-rostralanteriorcingulate	4.03
ctx-lh-cuneus	ctx-lh-rostralanteriorcingulate	3.93
ctx-lh-inferiorparietal	ctx-lh-rostralanteriorcingulate	4.15
ctx-lh-lateraloccipital	ctx-lh-rostralanteriorcingulate	5.01
ctx-lh-lateralorbitofrontal	ctx-lh-rostralanteriorcingulate	4.34
ctx-lh-lingual	ctx-lh-rostralanteriorcingulate	4.4
ctx-lh-paracentral	ctx-lh-rostralanteriorcingulate	7.28
ctx-lh-parsopercularis	ctx-lh-rostralanteriorcingulate	4.09
ctx-lh-parstriangularis	ctx-lh-rostralanteriorcingulate	3.78
ctx-lh-postcentral	ctx-lh-rostralanteriorcingulate	5.45
ctx-lh-precentral	ctx-lh-rostralanteriorcingulate	5.51
ctx-lh-precuneus	ctx-lh-rostralanteriorcingulate	5.1
ctx-lh-caudalanteriorcingulate	ctx-lh-rostralmiddlefrontal	3.18
ctx-lh-lateraloccipital	ctx-lh-rostralmiddlefrontal	6.04
ctx-lh-postcentral	ctx-lh-rostralmiddlefrontal	4.11
ctx-lh-lateraloccipital	ctx-lh-superiorfrontal	5.47
ctx-lh-precentral	ctx-lh-superiorfrontal	3.66
ctx-lh-precuneus	ctx-lh-superiorfrontal	3.25
ctx-lh-rostralanteriorcingulate	ctx-lh-superiorfrontal	3.69
ctx-lh-caudalanteriorcingulate	ctx-lh-superiorparietal	5.36
ctx-lh-caudalmiddlefrontal	ctx-lh-superiorparietal	5.34
ctx-lh-fusiform	ctx-lh-superiorparietal	4.44
ctx-lh-inferiorparietal	ctx-lh-superiorparietal	5.15
ctx-lh-inferiortemporal	ctx-lh-superiorparietal	6.12
ctx-lh-isthmuscingulate	ctx-lh-superiorparietal	3.85
ctx-lh-lateraloccipital	ctx-lh-superiorparietal	3.86
ctx-lh-lateralorbitofrontal	ctx-lh-superiorparietal	4.77
ctx-lh-medialorbitofrontal	ctx-lh-superiorparietal	5.99
ctx-lh-middletemporal	ctx-lh-superiorparietal	5.3
ctx-lh-parsopercularis	ctx-lh-superiorparietal	4.73
ctx-lh-parsorbitalis	ctx-lh-superiorparietal	4.37
ctx-lh-parstriangularis	ctx-lh-superiorparietal	3.97
ctx-lh-posteriorcingulate	ctx-lh-superiorparietal	4.2
ctx-lh-precuneus	ctx-lh-superiorparietal	4
ctx-lh-rostralanteriorcingulate	ctx-lh-superiorparietal	6.86
ctx-lh-rostralmiddlefrontal	ctx-lh-superiorparietal	5.64
ctx-lh-superiorfrontal	ctx-lh-superiorparietal	4.23
ctx-lh-bankssts	ctx-lh-superiortemporal	3.38
ctx-lh-fusiform	ctx-lh-supramarginal	3.27
ctx-lh-inferiortemporal	ctx-lh-supramarginal	4.29

ctx-lh-lateraloccipital	ctx-lh-supramarginal	6.37
ctx-lh-middletemporal	ctx-lh-supramarginal	3.2
ctx-lh-parsorbitalis	ctx-lh-supramarginal	3.71
ctx-lh-precuneus	ctx-lh-supramarginal	4.45
ctx-lh-rostralanteriorcingulate	ctx-lh-supramarginal	4.38
ctx-lh-superiorparietal	ctx-lh-supramarginal	6.31
ctx-lh-lateraloccipital	ctx-lh-frontalpole	4.12
ctx-lh-postcentral	ctx-lh-frontalpole	3.88
ctx-lh-superiorparietal	ctx-lh-frontalpole	3.86
ctx-lh-superiortemporal	ctx-lh-temporalpole	3.26
ctx-lh-fusiform	ctx-lh-transversetemporal	3.52
ctx-lh-inferiortemporal	ctx-lh-transversetemporal	5.16
ctx-lh-isthmuscingulate	ctx-lh-transversetemporal	4.1
ctx-lh-lateralorbitofrontal	ctx-lh-transversetemporal	3.29
ctx-lh-lingual	ctx-lh-transversetemporal	3.2
ctx-lh-medialorbitofrontal	ctx-lh-transversetemporal	3.19
ctx-lh-rostralanteriorcingulate	ctx-lh-transversetemporal	6.01
ctx-lh-frontalpole	ctx-lh-transversetemporal	4.61
ctx-lh-temporalpole	ctx-lh-transversetemporal	3.48
ctx-lh-bankssts	ctx-lh-insula	4.94
ctx-lh-caudalmiddlefrontal	ctx-lh-insula	5.23
ctx-lh-cuneus	ctx-lh-insula	4.43
ctx-lh-entorhinal	ctx-lh-insula	3.27
ctx-lh-fusiform	ctx-lh-insula	5.79
ctx-lh-inferiorparietal	ctx-lh-insula	6.43
ctx-lh-inferiortemporal	ctx-lh-insula	5.3
ctx-lh-isthmuscingulate	ctx-lh-insula	4.25
ctx-lh-lateraloccipital	ctx-lh-insula	5.33
ctx-lh-lateralorbitofrontal	ctx-lh-insula	4.86
ctx-lh-lingual	ctx-lh-insula	6.94
ctx-lh-middletemporal	ctx-lh-insula	5.22
ctx-lh-parahippocampal	ctx-lh-insula	4.39
ctx-lh-paracentral	ctx-lh-insula	7.14
ctx-lh-parsopercularis	ctx-lh-insula	5.02
ctx-lh-parsorbitalis	ctx-lh-insula	3.83
ctx-lh-parstriangularis	ctx-lh-insula	5.11
ctx-lh-pericalcarine	ctx-lh-insula	3.55
ctx-lh-postcentral	ctx-lh-insula	5.15
ctx-lh-precentral	ctx-lh-insula	6.47
ctx-lh-precuneus	ctx-lh-insula	6.74
ctx-lh-rostralanteriorcingulate	ctx-lh-insula	5.47
ctx-lh-rostralmiddlefrontal	ctx-lh-insula	4.39
ctx-lh-superiorfrontal	ctx-lh-insula	4.51
ctx-lh-superiorparietal	ctx-lh-insula	8.1
ctx-lh-superiortemporal	ctx-lh-insula	9.09
ctx-lh-supramarginal	ctx-lh-insula	5.35

ctx-lh-transversetemporal	ctx-lh-insula	4.85
ctx-lh-superiorparietal	ctx-rh-bankssts	3.78
ctx-lh-insula	ctx-rh-bankssts	5.44
ctx-lh-cuneus	ctx-rh-caudalanteriorcingulate	5.26
ctx-lh-lateraloccipital	ctx-rh-caudalanteriorcingulate	4.82
ctx-lh-lingual	ctx-rh-caudalanteriorcingulate	5.02
ctx-lh-paracentral	ctx-rh-caudalanteriorcingulate	3.55
ctx-lh-pericalcarine	ctx-rh-caudalanteriorcingulate	4.39
ctx-lh-postcentral	ctx-rh-caudalanteriorcingulate	7.01
ctx-lh-superiorparietal	ctx-rh-caudalanteriorcingulate	6.42
ctx-lh-inferiortemporal	ctx-rh-caudalmiddlefrontal	3.18
ctx-lh-lateraloccipital	ctx-rh-caudalmiddlefrontal	4.2
ctx-lh-precuneus	ctx-rh-caudalmiddlefrontal	4.29
ctx-lh-rostralanteriorcingulate	ctx-rh-caudalmiddlefrontal	4.56
ctx-lh-superiorparietal	ctx-rh-caudalmiddlefrontal	4.82
ctx-lh-insula	ctx-rh-caudalmiddlefrontal	5.83
ctx-lh-caudalanteriorcingulate	ctx-rh-cuneus	4.63
ctx-lh-inferiortemporal	ctx-rh-cuneus	3.52
ctx-lh-isthmuscingulate	ctx-rh-cuneus	3.81
ctx-lh-medialorbitofrontal	ctx-rh-cuneus	4.61
ctx-lh-posteriorcingulate	ctx-rh-cuneus	4.02
ctx-lh-rostralanteriorcingulate	ctx-rh-cuneus	4.43
ctx-lh-frontalpole	ctx-rh-cuneus	3.12
ctx-lh-insula	ctx-rh-cuneus	5.07
ctx-rh-caudalanteriorcingulate	ctx-rh-cuneus	5.44
ctx-lh-insula	ctx-rh-entorhinal	3.18
ctx-lh-paracentral	ctx-rh-fusiform	5.88
ctx-lh-precentral	ctx-rh-fusiform	4.99
ctx-lh-rostralanteriorcingulate	ctx-rh-fusiform	3.19
ctx-lh-superiorfrontal	ctx-rh-fusiform	3.47
ctx-lh-superiorparietal	ctx-rh-fusiform	4.25
ctx-lh-superiortemporal	ctx-rh-fusiform	3.85
ctx-lh-supramarginal	ctx-rh-fusiform	3.52
ctx-lh-transversetemporal	ctx-rh-fusiform	3.58
ctx-lh-insula	ctx-rh-fusiform	6.46
ctx-rh-caudalmiddlefrontal	ctx-rh-fusiform	3.6
ctx-lh-inferiortemporal	ctx-rh-inferiorparietal	3.5
ctx-lh-lateraloccipital	ctx-rh-inferiorparietal	4.45
ctx-lh-superiorparietal	ctx-rh-inferiorparietal	4.86
ctx-lh-supramarginal	ctx-rh-inferiorparietal	3.73
ctx-lh-insula	ctx-rh-inferiorparietal	5.92
ctx-lh-caudalmiddlefrontal	ctx-rh-inferiortemporal	3.46
ctx-lh-inferiorparietal	ctx-rh-inferiortemporal	3.46
ctx-lh-lateraloccipital	ctx-rh-inferiortemporal	4.65
ctx-lh-paracentral	ctx-rh-inferiortemporal	6.46
ctx-lh-parsopercularis	ctx-rh-inferiortemporal	3.44

ctx-lh-postcentral	ctx-rh-inferiortemporal	3.65
ctx-lh-precentral	ctx-rh-inferiortemporal	5.28
ctx-lh-precuneus	ctx-rh-inferiortemporal	4.15
ctx-lh-superiorfrontal	ctx-rh-inferiortemporal	3.14
ctx-lh-superiorparietal	ctx-rh-inferiortemporal	5.92
ctx-lh-supramarginal	ctx-rh-inferiortemporal	4.1
ctx-lh-transversetemporal	ctx-rh-inferiortemporal	5.66
ctx-lh-insula	ctx-rh-inferiortemporal	6.81
ctx-rh-caudalmiddlefrontal	ctx-rh-inferiortemporal	3.53
ctx-rh-inferiorparietal	ctx-rh-inferiortemporal	3.72
ctx-lh-cuneus	ctx-rh-isthmuscingulate	4.26
ctx-lh-lateraloccipital	ctx-rh-isthmuscingulate	3.53
ctx-lh-lingual	ctx-rh-isthmuscingulate	3.27
ctx-lh-paracentral	ctx-rh-isthmuscingulate	4.1
ctx-lh-postcentral	ctx-rh-isthmuscingulate	4.17
ctx-lh-superiorparietal	ctx-rh-isthmuscingulate	4.75
ctx-lh-frontalpole	ctx-rh-isthmuscingulate	3.38
ctx-lh-transversetemporal	ctx-rh-isthmuscingulate	3.26
ctx-lh-insula	ctx-rh-isthmuscingulate	3.71
ctx-rh-cuneus	ctx-rh-isthmuscingulate	4.81
ctx-lh-caudalanteriorcingulate	ctx-rh-lateraloccipital	5.35
ctx-lh-caudalmiddlefrontal	ctx-rh-lateraloccipital	6.27
ctx-lh-fusiform	ctx-rh-lateraloccipital	4.31
ctx-lh-inferiorparietal	ctx-rh-lateraloccipital	6.72
ctx-lh-inferiortemporal	ctx-rh-lateraloccipital	5.87
ctx-lh-lateralorbitofrontal	ctx-rh-lateraloccipital	5.12
ctx-lh-medialorbitofrontal	ctx-rh-lateraloccipital	6.45
ctx-lh-middletemporal	ctx-rh-lateraloccipital	4.27
ctx-lh-parahippocampal	ctx-rh-lateraloccipital	3.24
ctx-lh-paracentral	ctx-rh-lateraloccipital	5.5
ctx-lh-parsopercularis	ctx-rh-lateraloccipital	6.92
ctx-lh-parstriangularis	ctx-rh-lateraloccipital	5.48
ctx-lh-posteriorcingulate	ctx-rh-lateraloccipital	4.67
ctx-lh-precentral	ctx-rh-lateraloccipital	5.42
ctx-lh-precuneus	ctx-rh-lateraloccipital	4.72
ctx-lh-rostralanteriorcingulate	ctx-rh-lateraloccipital	4.69
ctx-lh-rostralmiddlefrontal	ctx-rh-lateraloccipital	7.43
ctx-lh-superiorfrontal	ctx-rh-lateraloccipital	7.12
ctx-lh-superiorparietal	ctx-rh-lateraloccipital	5.48
ctx-lh-supramarginal	ctx-rh-lateraloccipital	7.46
ctx-lh-frontalpole	ctx-rh-lateraloccipital	4.94
ctx-lh-transversetemporal	ctx-rh-lateraloccipital	3.77
ctx-lh-insula	ctx-rh-lateraloccipital	7.38
ctx-rh-caudalanteriorcingulate	ctx-rh-lateraloccipital	5.53
ctx-rh-caudalmiddlefrontal	ctx-rh-lateraloccipital	6.23
ctx-rh-fusiform	ctx-rh-lateraloccipital	3.54

ctx-rh-inferiorparietal	ctx-rh-lateraloccipital	6.29
ctx-rh-inferiortemporal	ctx-rh-lateraloccipital	4.83
ctx-lh-lateraloccipital	ctx-rh-lateralorbitofrontal	4.89
ctx-lh-lingual	ctx-rh-lateralorbitofrontal	3.2
ctx-lh-paracentral	ctx-rh-lateralorbitofrontal	4.43
ctx-lh-postcentral	ctx-rh-lateralorbitofrontal	3.78
ctx-lh-rostralanteriorcingulate	ctx-rh-lateralorbitofrontal	3.75
ctx-lh-superiorparietal	ctx-rh-lateralorbitofrontal	4.71
ctx-lh-transversetemporal	ctx-rh-lateralorbitofrontal	3.71
ctx-lh-insula	ctx-rh-lateralorbitofrontal	3.62
ctx-rh-cuneus	ctx-rh-lateralorbitofrontal	3.44
ctx-rh-lateraloccipital	ctx-rh-lateralorbitofrontal	4.81
ctx-lh-caudalanteriorcingulate	ctx-rh-lingual	3.95
ctx-lh-inferiortemporal	ctx-rh-lingual	3.33
ctx-lh-medialorbitofrontal	ctx-rh-lingual	3.51
ctx-lh-paracentral	ctx-rh-lingual	3.12
ctx-lh-posteriorcingulate	ctx-rh-lingual	3.86
ctx-lh-rostralanteriorcingulate	ctx-rh-lingual	4.53
ctx-lh-insula	ctx-rh-lingual	6.45
ctx-rh-caudalanteriorcingulate	ctx-rh-lingual	4.95
ctx-rh-isthmuscingulate	ctx-rh-lingual	3.49
ctx-rh-lateralorbitofrontal	ctx-rh-lingual	3.44
ctx-lh-cuneus	ctx-rh-medialorbitofrontal	4.12
ctx-lh-inferiorparietal	ctx-rh-medialorbitofrontal	3.42
ctx-lh-lateraloccipital	ctx-rh-medialorbitofrontal	7.7
ctx-lh-lingual	ctx-rh-medialorbitofrontal	4.72
ctx-lh-paracentral	ctx-rh-medialorbitofrontal	4.87
ctx-lh-pericalcarine	ctx-rh-medialorbitofrontal	4.53
ctx-lh-postcentral	ctx-rh-medialorbitofrontal	6.52
ctx-lh-precentral	ctx-rh-medialorbitofrontal	3.47
ctx-lh-precuneus	ctx-rh-medialorbitofrontal	3.83
ctx-lh-rostralmiddlefrontal	ctx-rh-medialorbitofrontal	3.41
ctx-lh-superiorparietal	ctx-rh-medialorbitofrontal	7.61
ctx-lh-transversetemporal	ctx-rh-medialorbitofrontal	4.4
ctx-rh-caudalanteriorcingulate	ctx-rh-medialorbitofrontal	3.22
ctx-rh-cuneus	ctx-rh-medialorbitofrontal	5.08
ctx-rh-lateraloccipital	ctx-rh-medialorbitofrontal	7.06
ctx-rh-lingual	ctx-rh-medialorbitofrontal	5.34
ctx-lh-inferiorparietal	ctx-rh-middletemporal	3.96
ctx-lh-lateraloccipital	ctx-rh-middletemporal	5.43
ctx-lh-paracentral	ctx-rh-middletemporal	4.73
ctx-lh-parsopercularis	ctx-rh-middletemporal	3.76
ctx-lh-precentral	ctx-rh-middletemporal	4.1
ctx-lh-precuneus	ctx-rh-middletemporal	4.51
ctx-lh-superiorfrontal	ctx-rh-middletemporal	3.58
ctx-lh-superiorparietal	ctx-rh-middletemporal	6

ctx-lh-superiortemporal	ctx-rh-middletemporal	3.86
ctx-lh-supramarginal	ctx-rh-middletemporal	3.9
ctx-lh-transversetemporal	ctx-rh-middletemporal	3.43
ctx-lh-insula	ctx-rh-middletemporal	5.24
ctx-rh-inferiorparietal	ctx-rh-middletemporal	3.94
ctx-rh-lateraloccipital	ctx-rh-middletemporal	5.46
ctx-lh-lateraloccipital	ctx-rh-parahippocampal	3.25
ctx-lh-paracentral	ctx-rh-parahippocampal	3.53
ctx-lh-insula	ctx-rh-parahippocampal	4.76
ctx-rh-lateraloccipital	ctx-rh-parahippocampal	3.21
ctx-lh-caudalanteriorcingulate	ctx-rh-paracentral	3.64
ctx-lh-fusiform	ctx-rh-paracentral	5.28
ctx-lh-inferiortemporal	ctx-rh-paracentral	5.5
ctx-lh-lateralorbitofrontal	ctx-rh-paracentral	4.26
ctx-lh-middletemporal	ctx-rh-paracentral	4.12
ctx-lh-parahippocampal	ctx-rh-paracentral	3.87
ctx-lh-rostralanteriorcingulate	ctx-rh-paracentral	5.72
ctx-lh-superiorfrontal	ctx-rh-paracentral	4.04
ctx-lh-frontalpole	ctx-rh-paracentral	3.23
ctx-lh-insula	ctx-rh-paracentral	7
ctx-rh-fusiform	ctx-rh-paracentral	5.95
ctx-rh-inferiortemporal	ctx-rh-paracentral	5.89
ctx-rh-isthmuscingulate	ctx-rh-paracentral	3.6
ctx-rh-lateraloccipital	ctx-rh-paracentral	4.53
ctx-rh-medialorbitofrontal	ctx-rh-paracentral	4.38
ctx-rh-middletemporal	ctx-rh-paracentral	4.97
ctx-rh-parahippocampal	ctx-rh-paracentral	3.23
ctx-lh-inferiortemporal	ctx-rh-parsopercularis	3.14
ctx-lh-lateraloccipital	ctx-rh-parsopercularis	4.13
ctx-lh-middletemporal	ctx-rh-parsopercularis	3.25
ctx-lh-parsorbitalis	ctx-rh-parsopercularis	3.43
ctx-lh-precuneus	ctx-rh-parsopercularis	3.54
ctx-lh-rostralanteriorcingulate	ctx-rh-parsopercularis	4.91
ctx-lh-superiorparietal	ctx-rh-parsopercularis	3.53
ctx-lh-supramarginal	ctx-rh-parsopercularis	3.59
ctx-lh-insula	ctx-rh-parsopercularis	4.63
ctx-rh-fusiform	ctx-rh-parsopercularis	3.19
ctx-rh-inferiortemporal	ctx-rh-parsopercularis	3.76
ctx-rh-lateraloccipital	ctx-rh-parsopercularis	5.19
ctx-rh-middletemporal	ctx-rh-parsopercularis	4.06
ctx-lh-caudalanteriorcingulate	ctx-rh-parsorbitalis	3.12
ctx-lh-lateraloccipital	ctx-rh-parsorbitalis	4.87
ctx-lh-paracentral	ctx-rh-parsorbitalis	4.01
ctx-lh-postcentral	ctx-rh-parsorbitalis	4.19
ctx-lh-superiorparietal	ctx-rh-parsorbitalis	5.03
ctx-lh-transversetemporal	ctx-rh-parsorbitalis	4.47

ctx-lh-insula	ctx-rh-parsorbitalis	4
ctx-rh-lateraloccipital	ctx-rh-parsorbitalis	4.86
ctx-rh-paracentral	ctx-rh-parsorbitalis	4.21
ctx-rh-parsopercularis	ctx-rh-parsorbitalis	3.11
ctx-lh-lateraloccipital	ctx-rh-parstriangularis	4.18
ctx-lh-rostralanteriorcingulate	ctx-rh-parstriangularis	3.53
ctx-lh-superiorparietal	ctx-rh-parstriangularis	4.25
ctx-lh-insula	ctx-rh-parstriangularis	4.85
ctx-rh-lateraloccipital	ctx-rh-parstriangularis	5.24
ctx-lh-insula	ctx-rh-pericalcarine	3.51
ctx-rh-caudalanteriorcingulate	ctx-rh-pericalcarine	3.85
ctx-rh-medialorbitofrontal	ctx-rh-pericalcarine	3.93
ctx-lh-caudalanteriorcingulate	ctx-rh-postcentral	4.85
ctx-lh-inferiortemporal	ctx-rh-postcentral	4.37
ctx-lh-isthmuscingulate	ctx-rh-postcentral	4.64
ctx-lh-lateralorbitofrontal	ctx-rh-postcentral	3.75
ctx-lh-medialorbitofrontal	ctx-rh-postcentral	5.19
ctx-lh-parahippocampal	ctx-rh-postcentral	3.98
ctx-lh-posteriorcingulate	ctx-rh-postcentral	4.57
ctx-lh-rostralanteriorcingulate	ctx-rh-postcentral	5.59
ctx-lh-rostralmiddlefrontal	ctx-rh-postcentral	5.11
ctx-lh-frontalpole	ctx-rh-postcentral	4.05
ctx-lh-insula	ctx-rh-postcentral	6.12
ctx-rh-caudalanteriorcingulate	ctx-rh-postcentral	8.1
ctx-rh-inferiortemporal	ctx-rh-postcentral	4.33
ctx-rh-isthmuscingulate	ctx-rh-postcentral	5.91
ctx-rh-lateralorbitofrontal	ctx-rh-postcentral	4.94
ctx-rh-medialorbitofrontal	ctx-rh-postcentral	7.25
ctx-rh-parahippocampal	ctx-rh-postcentral	4
ctx-rh-parsorbitalis	ctx-rh-postcentral	5.03
ctx-lh-lingual	ctx-rh-posteriorcingulate	3.8
ctx-lh-postcentral	ctx-rh-posteriorcingulate	3.8
ctx-lh-superiorparietal	ctx-rh-posteriorcingulate	3.92
ctx-rh-cuneus	ctx-rh-posteriorcingulate	3.36
ctx-rh-lateraloccipital	ctx-rh-posteriorcingulate	3.86
ctx-rh-postcentral	ctx-rh-posteriorcingulate	5.23
ctx-lh-fusiform	ctx-rh-precentral	4.96
ctx-lh-inferiortemporal	ctx-rh-precentral	6.31
ctx-lh-lateraloccipital	ctx-rh-precentral	3.2
ctx-lh-lateralorbitofrontal	ctx-rh-precentral	4.22
ctx-lh-middletemporal	ctx-rh-precentral	4.39
ctx-lh-parsorbitalis	ctx-rh-precentral	3.56
ctx-lh-rostralanteriorcingulate	ctx-rh-precentral	6.53
ctx-lh-superiorfrontal	ctx-rh-precentral	4.92
ctx-lh-insula	ctx-rh-precentral	7.52
ctx-rh-caudalanteriorcingulate	ctx-rh-precentral	3.28

ctx-rh-fusiform	ctx-rh-precentral	5.59
ctx-rh-inferiortemporal	ctx-rh-precentral	5.96
ctx-rh-lateraloccipital	ctx-rh-precentral	4.96
ctx-rh-medialorbitofrontal	ctx-rh-precentral	3.77
ctx-rh-middletemporal	ctx-rh-precentral	5.26
ctx-rh-parsorbitalis	ctx-rh-precentral	3.38
ctx-lh-inferiortemporal	ctx-rh-precuneus	3.83
ctx-lh-rostralanteriorcingulate	ctx-rh-precuneus	4.89
ctx-lh-insula	ctx-rh-precuneus	7.62
ctx-rh-fusiform	ctx-rh-precuneus	3.53
ctx-rh-inferiortemporal	ctx-rh-precuneus	4.16
ctx-rh-lateraloccipital	ctx-rh-precuneus	4.31
ctx-rh-medialorbitofrontal	ctx-rh-precuneus	3.41
ctx-rh-middletemporal	ctx-rh-precuneus	4.3
ctx-lh-caudalmiddlefrontal	ctx-rh-rostralanteriorcingulate	4.04
ctx-lh-cuneus	ctx-rh-rostralanteriorcingulate	6.1
ctx-lh-inferiorparietal	ctx-rh-rostralanteriorcingulate	5.08
ctx-lh-isthmuscingulate	ctx-rh-rostralanteriorcingulate	4.4
ctx-lh-lateraloccipital	ctx-rh-rostralanteriorcingulate	7.49
ctx-lh-lingual	ctx-rh-rostralanteriorcingulate	6.57
ctx-lh-paracentral	ctx-rh-rostralanteriorcingulate	7.05
ctx-lh-parsopercularis	ctx-rh-rostralanteriorcingulate	3.83
ctx-lh-parstriangularis	ctx-rh-rostralanteriorcingulate	5.93
ctx-lh-pericalcarine	ctx-rh-rostralanteriorcingulate	5.36
ctx-lh-postcentral	ctx-rh-rostralanteriorcingulate	7.23
ctx-lh-precentral	ctx-rh-rostralanteriorcingulate	5.12
ctx-lh-precuneus	ctx-rh-rostralanteriorcingulate	5.6
ctx-lh-rostralmiddlefrontal	ctx-rh-rostralanteriorcingulate	4.47
ctx-lh-superiorfrontal	ctx-rh-rostralanteriorcingulate	3.1
ctx-lh-superiorparietal	ctx-rh-rostralanteriorcingulate	8.17
ctx-lh-supramarginal	ctx-rh-rostralanteriorcingulate	4.62
ctx-lh-transversetemporal	ctx-rh-rostralanteriorcingulate	5.15
ctx-rh-caudalmiddlefrontal	ctx-rh-rostralanteriorcingulate	5.38
ctx-rh-cuneus	ctx-rh-rostralanteriorcingulate	6.74
ctx-rh-inferiorparietal	ctx-rh-rostralanteriorcingulate	3.75
ctx-rh-isthmuscingulate	ctx-rh-rostralanteriorcingulate	3.34
ctx-rh-lateraloccipital	ctx-rh-rostralanteriorcingulate	7.38
ctx-rh-lingual	ctx-rh-rostralanteriorcingulate	6.3
ctx-rh-medialorbitofrontal	ctx-rh-rostralanteriorcingulate	3.71
ctx-rh-paracentral	ctx-rh-rostralanteriorcingulate	5.96
ctx-rh-parsopercularis	ctx-rh-rostralanteriorcingulate	4.58
ctx-rh-parsorbitalis	ctx-rh-rostralanteriorcingulate	3.22
ctx-rh-parstriangularis	ctx-rh-rostralanteriorcingulate	5.33
ctx-rh-pericalcarine	ctx-rh-rostralanteriorcingulate	5.33
ctx-rh-postcentral	ctx-rh-rostralanteriorcingulate	7.86
ctx-rh-precentral	ctx-rh-rostralanteriorcingulate	6.44

ctx-rh-precuneus	ctx-rh-rostralanteriorcingulate	5.3
ctx-lh-lateraloccipital	ctx-rh-rostralmiddlefrontal	4.79
ctx-lh-postcentral	ctx-rh-rostralmiddlefrontal	3.28
ctx-lh-superiorparietal	ctx-rh-rostralmiddlefrontal	4.82
ctx-lh-insula	ctx-rh-rostralmiddlefrontal	3.78
ctx-rh-lateraloccipital	ctx-rh-rostralmiddlefrontal	5.86
ctx-rh-postcentral	ctx-rh-rostralmiddlefrontal	3.79
ctx-rh-rostralanteriorcingulate	ctx-rh-rostralmiddlefrontal	4.84
ctx-lh-lateraloccipital	ctx-rh-superiorfrontal	6.11
ctx-lh-middletemporal	ctx-rh-superiorfrontal	3.41
ctx-lh-paracentral	ctx-rh-superiorfrontal	3.33
ctx-lh-precuneus	ctx-rh-superiorfrontal	4.18
ctx-lh-rostralanteriorcingulate	ctx-rh-superiorfrontal	3.7
ctx-lh-superiorparietal	ctx-rh-superiorfrontal	5.5
ctx-lh-insula	ctx-rh-superiorfrontal	4.79
ctx-rh-fusiform	ctx-rh-superiorfrontal	3.77
ctx-rh-inferiortemporal	ctx-rh-superiorfrontal	3.34
ctx-rh-lateraloccipital	ctx-rh-superiorfrontal	7.98
ctx-rh-middletemporal	ctx-rh-superiorfrontal	3.83
ctx-rh-paracentral	ctx-rh-superiorfrontal	4.3
ctx-rh-parsopercularis	ctx-rh-superiorfrontal	3.64
ctx-rh-postcentral	ctx-rh-superiorfrontal	3.33
ctx-rh-precentral	ctx-rh-superiorfrontal	4.81
ctx-rh-precuneus	ctx-rh-superiorfrontal	3.25
ctx-rh-rostralanteriorcingulate	ctx-rh-superiorfrontal	3.97
ctx-lh-caudalanteriorcingulate	ctx-rh-superiorparietal	5.01
ctx-lh-caudalmiddlefrontal	ctx-rh-superiorparietal	4.29
ctx-lh-fusiform	ctx-rh-superiorparietal	3.82
ctx-lh-inferiorparietal	ctx-rh-superiorparietal	3.63
ctx-lh-inferiortemporal	ctx-rh-superiorparietal	5.19
ctx-lh-lateralorbitofrontal	ctx-rh-superiorparietal	4.23
ctx-lh-medialorbitofrontal	ctx-rh-superiorparietal	5.45
ctx-lh-middletemporal	ctx-rh-superiorparietal	4.73
ctx-lh-parsopercularis	ctx-rh-superiorparietal	3.93
ctx-lh-parsorbitalis	ctx-rh-superiorparietal	4.24
ctx-lh-parstriangularis	ctx-rh-superiorparietal	3.49
ctx-lh-posteriorcingulate	ctx-rh-superiorparietal	3.63
ctx-lh-rostralanteriorcingulate	ctx-rh-superiorparietal	6.2
ctx-lh-rostralmiddlefrontal	ctx-rh-superiorparietal	5.77
ctx-lh-superiorfrontal	ctx-rh-superiorparietal	3.78
ctx-lh-supramarginal	ctx-rh-superiorparietal	5.54
ctx-lh-frontalpole	ctx-rh-superiorparietal	4.2
ctx-lh-insula	ctx-rh-superiorparietal	7.54
ctx-rh-caudalanteriorcingulate	ctx-rh-superiorparietal	6.52
ctx-rh-caudalmiddlefrontal	ctx-rh-superiorparietal	4.12
ctx-rh-inferiorparietal	ctx-rh-superiorparietal	4.28

ctx-rh-inferiortemporal	ctx-rh-superiorparietal	5.2
ctx-rh-isthmuscingulate	ctx-rh-superiorparietal	3.82
ctx-rh-lateraloccipital	ctx-rh-superiorparietal	5.05
ctx-rh-lateralorbitofrontal	ctx-rh-superiorparietal	4.59
ctx-rh-medialorbitofrontal	ctx-rh-superiorparietal	6.97
ctx-rh-middletemporal	ctx-rh-superiorparietal	5.55
ctx-rh-parsopercularis	ctx-rh-superiorparietal	3.13
ctx-rh-parsorbitalis	ctx-rh-superiorparietal	5.02
ctx-rh-parstriangularis	ctx-rh-superiorparietal	3.43
ctx-rh-posteriorcingulate	ctx-rh-superiorparietal	3.65
ctx-rh-rostralanteriorcingulate	ctx-rh-superiorparietal	7.85
ctx-rh-rostralmiddlefrontal	ctx-rh-superiorparietal	4.6
ctx-rh-superiorfrontal	ctx-rh-superiorparietal	5.07
ctx-lh-bankssts	ctx-rh-superiortemporal	3.46
ctx-lh-insula	ctx-rh-superiortemporal	7.97
ctx-rh-fusiform	ctx-rh-superiortemporal	3.76
ctx-lh-inferiorparietal	ctx-rh-supramarginal	3.96
ctx-lh-inferiortemporal	ctx-rh-supramarginal	3.44
ctx-lh-lateraloccipital	ctx-rh-supramarginal	5.96
ctx-lh-middletemporal	ctx-rh-supramarginal	3.22
ctx-lh-parsopercularis	ctx-rh-supramarginal	3.49
ctx-lh-parsorbitalis	ctx-rh-supramarginal	4.17
ctx-lh-precuneus	ctx-rh-supramarginal	4.04
ctx-lh-rostralanteriorcingulate	ctx-rh-supramarginal	4.15
ctx-lh-superiorparietal	ctx-rh-supramarginal	5.33
ctx-lh-supramarginal	ctx-rh-supramarginal	3.13
ctx-lh-insula	ctx-rh-supramarginal	4.56
ctx-rh-caudalmiddlefrontal	ctx-rh-supramarginal	3.75
ctx-rh-fusiform	ctx-rh-supramarginal	3.32
ctx-rh-inferiorparietal	ctx-rh-supramarginal	4.49
ctx-rh-inferiortemporal	ctx-rh-supramarginal	3.79
ctx-rh-lateraloccipital	ctx-rh-supramarginal	6.91
ctx-rh-middletemporal	ctx-rh-supramarginal	4.4
ctx-rh-parsopercularis	ctx-rh-supramarginal	3.39
ctx-rh-rostralanteriorcingulate	ctx-rh-supramarginal	4.21
ctx-rh-superiorfrontal	ctx-rh-supramarginal	3.15
ctx-rh-superiorparietal	ctx-rh-supramarginal	4.77
ctx-lh-postcentral	ctx-rh-frontalpole	3.38
ctx-lh-precentral	ctx-rh-frontalpole	3.74
ctx-lh-transversetemporal	ctx-rh-frontalpole	4.83
ctx-lh-insula	ctx-rh-frontalpole	3.1
ctx-rh-lateraloccipital	ctx-rh-frontalpole	3.12
ctx-rh-paracentral	ctx-rh-frontalpole	3.27
ctx-rh-parsopercularis	ctx-rh-frontalpole	3.36
ctx-rh-postcentral	ctx-rh-frontalpole	3.25
ctx-rh-precentral	ctx-rh-frontalpole	3.22

ctx-rh-supramarginal	ctx-rh-frontalpole	3.66
ctx-lh-transversetemporal	ctx-rh-temporalpole	3.2
ctx-lh-fusiform	ctx-rh-transversetemporal	3.43
ctx-lh-inferiortemporal	ctx-rh-transversetemporal	4.8
ctx-lh-isthmuscingulate	ctx-rh-transversetemporal	5.68
ctx-lh-lateralorbitofrontal	ctx-rh-transversetemporal	3.87
ctx-lh-lingual	ctx-rh-transversetemporal	4.37
ctx-lh-medialorbitofrontal	ctx-rh-transversetemporal	3.47
ctx-lh-parsorbitalis	ctx-rh-transversetemporal	3.5
ctx-lh-posteriorcingulate	ctx-rh-transversetemporal	3.43
ctx-lh-rostralanteriorcingulate	ctx-rh-transversetemporal	6.44
ctx-lh-superiortemporal	ctx-rh-transversetemporal	3.17
ctx-lh-frontalpole	ctx-rh-transversetemporal	4.3
ctx-lh-temporalpole	ctx-rh-transversetemporal	3.47
ctx-lh-insula	ctx-rh-transversetemporal	5.55
ctx-rh-caudalanteriorcingulate	ctx-rh-transversetemporal	3.72
ctx-rh-fusiform	ctx-rh-transversetemporal	3.4
ctx-rh-inferiortemporal	ctx-rh-transversetemporal	4.95
ctx-rh-isthmuscingulate	ctx-rh-transversetemporal	4.38
ctx-rh-lateraloccipital	ctx-rh-transversetemporal	4.73
ctx-rh-lateralorbitofrontal	ctx-rh-transversetemporal	4.07
ctx-rh-lingual	ctx-rh-transversetemporal	3.73
ctx-rh-medialorbitofrontal	ctx-rh-transversetemporal	5.08
ctx-rh-middletemporal	ctx-rh-transversetemporal	3.17
ctx-rh-parsorbitalis	ctx-rh-transversetemporal	4.74
ctx-rh-posteriorcingulate	ctx-rh-transversetemporal	3.22
ctx-rh-rostralanteriorcingulate	ctx-rh-transversetemporal	5.69
ctx-rh-frontalpole	ctx-rh-transversetemporal	5.14
ctx-rh-temporalpole	ctx-rh-transversetemporal	3.54
ctx-lh-bankssts	ctx-rh-insula	3.5
ctx-lh-caudalmiddlefrontal	ctx-rh-insula	3.68
ctx-lh-cuneus	ctx-rh-insula	3.3
ctx-lh-fusiform	ctx-rh-insula	3.89
ctx-lh-inferiorparietal	ctx-rh-insula	4.33
ctx-lh-inferiortemporal	ctx-rh-insula	4.08
ctx-lh-isthmuscingulate	ctx-rh-insula	3.12
ctx-lh-lateraloccipital	ctx-rh-insula	4.12
ctx-lh-lateralorbitofrontal	ctx-rh-insula	3.66
ctx-lh-lingual	ctx-rh-insula	5.39
ctx-lh-middletemporal	ctx-rh-insula	3.54
ctx-lh-paracentral	ctx-rh-insula	4.88
ctx-lh-parsopercularis	ctx-rh-insula	3.61
ctx-lh-parstriangularis	ctx-rh-insula	3.67
ctx-lh-postcentral	ctx-rh-insula	3.85
ctx-lh-precentral	ctx-rh-insula	4.41
ctx-lh-precuneus	ctx-rh-insula	4.33

ctx-lh-rostralanteriorcingulate	ctx-rh-insula	4.22
ctx-lh-superiorparietal	ctx-rh-insula	6.09
ctx-lh-superiortemporal	ctx-rh-insula	6.08
ctx-lh-supramarginal	ctx-rh-insula	3.54
ctx-rh-bankssts	ctx-rh-insula	3.74
ctx-rh-caudalmiddlefrontal	ctx-rh-insula	4.27
ctx-rh-cuneus	ctx-rh-insula	4.24
ctx-rh-fusiform	ctx-rh-insula	4.32
ctx-rh-inferiorparietal	ctx-rh-insula	4.32
ctx-rh-inferiortemporal	ctx-rh-insula	5.36
ctx-rh-lateraloccipital	ctx-rh-insula	5.97
ctx-rh-lingual	ctx-rh-insula	5.27
ctx-rh-middletemporal	ctx-rh-insula	3.57
ctx-rh-parahippocampal	ctx-rh-insula	3.5
ctx-rh-paracentral	ctx-rh-insula	4.52
ctx-rh-parsopercularis	ctx-rh-insula	3.85
ctx-rh-parstriangularis	ctx-rh-insula	3.53
ctx-rh-postcentral	ctx-rh-insula	4.52
ctx-rh-precentral	ctx-rh-insula	5.04
ctx-rh-precuneus	ctx-rh-insula	5.18
ctx-rh-superiorfrontal	ctx-rh-insula	3.21
ctx-rh-superiorparietal	ctx-rh-insula	5.74
ctx-rh-superiortemporal	ctx-rh-insula	5.89
ctx-rh-supramarginal	ctx-rh-insula	3.32
ctx-rh-frontalpole	ctx-rh-insula	3.16
ctx-rh-temporalpole	ctx-rh-insula	3.26

Network-based statistics, non-parametric permutation two-tailed t-tests FDR correction at a $P<0.05$
FDR, false discovery rate; ctx, cortex; lh, left hemisphere; mHD, manifest Huntington's disease; rh, right hemisphere.

Table S5 Network-based statistics analysis, significant connections (mHD > controls) [P_{FWE}=0.022]

Connection 1	Connection 2	Test Statistic
ctx-lh-lateraloccipital	ctx-lh-pericalcarine	4.79
ctx-lh-pericalcarine	ctx-rh-lateraloccipital	4.79
ctx-lh-lateraloccipital	ctx-rh-pericalcarine	4.77
ctx-rh-lateraloccipital	ctx-rh-pericalcarine	4.61
ctx-rh-pericalcarine	ctx-rh-superiorparietal	3.29

Network-based statistics, non-parametric permutation two-tailed t-tests FDR correction at a P<0.05
FDR, false discovery rate; ctx, cortex; lh, left hemisphere; mHD, manifest Huntington's disease; rh, right hemisphere.

Table S6 Network-based statistics analysis, significant correlations between connections and NfL in late preHD [P_{FWE}=0.044]

Connection 1	Connection 2	Test statistic
ctx-lh-parsopercularis	ctx-rh-parsopercularis	3.67
ctx-lh-parstriangularis	ctx-rh-parsopercularis	3.24
ctx-lh-supramarginal	ctx-rh-parsopercularis	3.7
ctx-rh-caudalmiddlefrontal	ctx-rh-parsopercularis	3.25
ctx-rh-lateraloccipital	ctx-rh-parsopercularis	3.31
ctx-rh-parsopercularis	ctx-rh-supramarginal	3.44

Network-based statistics, non-parametric permutation two-tailed t-tests FDR correction at a P<0.05

FDR, false discovery rate; ctx, cortex; lh, left hemisphere; late preHD, late premanifest Huntington's disease, NfL, neurofilament, rh, right hemisphere.

Table S7 Network-based statistics analysis, significant correlations between connections and NfL in mHD [P_{FWE}<0.0001]

Connection 1	Connection 2	Test statistic
ctx-lh-caudalanteriorcingulate	ctx-lh-cuneus	3.47
ctx-lh-cuneus	ctx-lh-parahippocampal	3.43
ctx-lh-fusiform	ctx-lh-parahippocampal	3.76
ctx-lh-lateraloccipital	ctx-lh-parahippocampal	3.72
ctx-lh-parahippocampal	ctx-lh-paracentral	3.36
ctx-lh-isthmuscingulate	ctx-lh-posteriorcingulate	3.33
ctx-lh-parahippocampal	ctx-lh-precuneus	3.41
ctx-lh-cuneus	ctx-lh-rostralanteriorcingulate	3.21
ctx-lh-lateraloccipital	ctx-lh-rostralmiddlefrontal	3.39
ctx-lh-parahippocampal	ctx-lh-supramarginal	3.15
ctx-lh-inferiorparietal	ctx-lh-frontalpole	3.14
ctx-lh-inferiortemporal	ctx-lh-frontalpole	3.62
ctx-lh-precentral	ctx-lh-frontalpole	3.36
ctx-lh-superiorfrontal	ctx-lh-frontalpole	3.5
ctx-lh-superiortemporal	ctx-lh-frontalpole	3.36
ctx-lh-frontalpole	ctx-lh-transversetemporal	3.95
ctx-lh-frontalpole	ctx-lh-insula	3.16
ctx-lh-superiorfrontal	ctx-rh-fusiform	3.53
ctx-lh-frontalpole	ctx-rh-fusiform	3.53
ctx-lh-frontalpole	ctx-rh-inferiorparietal	3.11
ctx-lh-lateraloccipital	ctx-rh-inferiortemporal	3.35
ctx-lh-paracentral	ctx-rh-inferiortemporal	3.59
ctx-lh-precentral	ctx-rh-inferiortemporal	3.48
ctx-rh-caudalmiddlefrontal	ctx-rh-inferiortemporal	3.34
ctx-lh-lateraloccipital	ctx-rh-lateralorbitofrontal	3.8
ctx-lh-paracentral	ctx-rh-lateralorbitofrontal	3.74
ctx-lh-parahippocampal	ctx-rh-lingual	3.47
ctx-lh-cuneus	ctx-rh-medialorbitofrontal	3.72
ctx-lh-isthmuscingulate	ctx-rh-medialorbitofrontal	3.11
ctx-lh-paracentral	ctx-rh-parahippocampal	3.21
ctx-lh-frontalpole	ctx-rh-paracentral	3.18
ctx-rh-caudalmiddlefrontal	ctx-rh-paracentral	3.17
ctx-rh-inferiortemporal	ctx-rh-paracentral	3.37
ctx-rh-parahippocampal	ctx-rh-paracentral	3.26
ctx-rh-inferiortemporal	ctx-rh-parsopercularis	3.4

ctx-rh-middletemporal	ctx-rh-parsopercularis	3.71
ctx-rh-inferiortemporal	ctx-rh-parstriangularis	3.16
ctx-rh-middletemporal	ctx-rh-parstriangularis	3.13
ctx-lh-caudalanteriorcingulate	ctx-rh-postcentral	3.44
ctx-lh-parahippocampal	ctx-rh-precentral	3.64
ctx-rh-inferiortemporal	ctx-rh-precentral	3.33
ctx-rh-parahippocampal	ctx-rh-precentral	3.11
ctx-lh-parahippocampal	ctx-rh-precuneus	3.51
ctx-rh-inferiortemporal	ctx-rh-precuneus	3.3
ctx-rh-middletemporal	ctx-rh-precuneus	3.99
ctx-lh-cuneus	ctx-rh-rostralmiddlefrontal	3.36
ctx-lh-lateraloccipital	ctx-rh-rostralmiddlefrontal	3.16
ctx-lh-frontalpole	ctx-rh-superiorfrontal	3.6
ctx-lh-parahippocampal	ctx-rh-superiorparietal	3.16
ctx-rh-inferiortemporal	ctx-rh-superiorparietal	3.43
ctx-rh-middletemporal	ctx-rh-superiorparietal	3.45
ctx-rh-parsorbitalis	ctx-rh-superiorparietal	3.61
ctx-rh-rostralmiddlefrontal	ctx-rh-superiorparietal	3.28
ctx-lh-parahippocampal	ctx-rh-supramarginal	3.56
ctx-lh-frontalpole	ctx-rh-supramarginal	3.27
ctx-rh-parahippocampal	ctx-rh-supramarginal	3.11
ctx-lh-paracentral	ctx-rh-insula	3.41
ctx-rh-lingual	ctx-rh-insula	3.62

Network-based statistics, non-parametric permutation two-tailed tests, FDR correction at a P<0.05

FDR, false discovery rate; Ctx, cortex; lh, left hemisphere; mHD, manifest Huntington's disease, NfL, neurofilament, rh, right hemisphere.

Table S8 Permutation analysis of nodal strength in early preHD

Regions	P_{FDR}	$P_{uncorrected}$	early preHD < controls	early preHD > controls	Difference
lh bankssts	0.81023478	0.3718	0.1902	0.8098	-0.2063815
lh caudalanteriorcingulate	0.81023478	0.5164	0.2613	0.7387	-0.090052
lh caudalmiddlefrontal	0.95447273	0.9264	0.4625	0.5375	-0.0123674
lh cuneus	0.81023478	0.1836	0.9102	0.0898	0.22385046
lh entorhinal	0.81023478	0.3042	0.8522	0.1478	0.16427168
lh fusiform	0.81023478	0.1426	0.9281	0.0719	0.22584056
lh inferiorparietal	0.81023478	0.4324	0.7861	0.2139	0.09426784
lh inferiortemporal	0.88354667	0.7399	0.3627	0.6373	-0.0564357
lh isthmuscingulate	0.81023478	0.5481	0.2753	0.7247	-0.0904087
lh lateraloccipital	0.81023478	0.1966	0.9036	0.0964	0.21176678
lh lateralorbitofrontal	0.9250125	0.8706	0.4304	0.5696	-0.0228976
lh lingual	0.068	0.001	0.9993	0.0007	0.56785742
lh medialorbitofrontal	0.81023478	0.1403	0.9282	0.0718	0.21731427
lh middletemporal	0.88354667	0.7089	0.6424	0.3576	0.0564195
lh parahippocampal	0.81023478	0.541	0.7325	0.2675	0.08914403
lh paracentral	0.9758	0.9758	0.5034	0.4966	0.00351123
lh parsopercularis	0.81023478	0.3034	0.8494	0.1506	0.13643077
lh parsorbitalis	0.81023478	0.1613	0.9166	0.0834	0.16596876
lh parstriangularis	0.95447273	0.9178	0.5387	0.4613	0.01400821
lh pericalcarine	0.88354667	0.7796	0.6167	0.3833	0.0526805
lh postcentral	0.9020254	0.8357	0.5814	0.4186	0.03283486
lh posteriorcingulate	0.81023478	0.1372	0.0702	0.9298	-0.2389626
lh precentral	0.88354667	0.7794	0.3975	0.6025	-0.0435773
lh precuneus	0.88354667	0.7356	0.6306	0.3694	0.04277028
lh rostralanteriorcingulate	0.81023478	0.3162	0.1575	0.8425	-0.1582197
lh rostralmiddlefrontal	0.85769811	0.6414	0.3079	0.6921	-0.0702449
lh superiorfrontal	0.81023478	0.0479	0.9768	0.0232	0.2677669
lh superiorparietal	0.85311667	0.6022	0.7027	0.2973	0.08042223
lh superiortemporal	0.81023478	0.1933	0.903	0.097	0.21102458
lh supramarginal	0.81023478	0.2595	0.8673	0.1327	0.14272285
lh frontalpole	0.85769811	0.6575	0.6741	0.3259	0.04090134
lh temporalpole	0.82236596	0.5684	0.7127	0.2873	0.05723896
lh transversetemporal	0.85769811	0.6183	0.6956	0.3044	0.06600138
lh insula	0.81023478	0.2854	0.1457	0.8543	-0.1267512
rh bankssts	0.85769811	0.6685	0.3383	0.6617	-0.0856551
rh caudalanteriorcingulate	0.81023478	0.4924	0.2504	0.7496	-0.0973208
rh caudalmiddlefrontal	0.81023478	0.4837	0.7548	0.2452	0.09185337
rh cuneus	0.81023478	0.1457	0.9296	0.0704	0.2256745
rh entorhinal	0.81023478	0.4766	0.2404	0.7596	-0.1325627
rh fusiform	0.85769811	0.6564	0.6698	0.3302	0.06604009
rh inferiorparietal	0.81023478	0.3992	0.8007	0.1993	0.09800603

rh inferiortemporal	0.81023478	0.4245	0.7873	0.2127	0.12849275
rh isthmuscingulate	0.9020254	0.829	0.5836	0.4164	0.03370862
rh lateraloccipital	0.81023478	0.0367	0.9824	0.0176	0.29457248
rh lateralorbitofrontal	0.81023478	0.3859	0.8041	0.1959	0.10090139
rh lingual	0.81023478	0.112	0.9427	0.0573	0.23602083
rh medialorbitofrontal	0.81023478	0.4952	0.7522	0.2478	0.09291189
rh middletemporal	0.81023478	0.5278	0.7324	0.2676	0.09516488
rh parahippocampal	0.89681967	0.8045	0.5912	0.4088	0.03727251
rh paracentral	0.81023478	0.4853	0.7555	0.2445	0.09113246
rh parsopercularis	0.81023478	0.4389	0.7812	0.2188	0.12168421
rh parsorbitalis	0.81023478	0.2635	0.8675	0.1325	0.12060769
rh parstriangularis	0.81023478	0.2582	0.8687	0.1313	0.14558603
rh pericalcarine	0.81023478	0.2414	0.8822	0.1178	0.24242477
rh postcentral	0.81023478	0.4003	0.1947	0.8053	-0.1300692
rh posteriorcingulate	0.81023478	0.0604	0.0303	0.9697	-0.2626291
rh precentral	0.81023478	0.4373	0.2201	0.7799	-0.1084722
rh precuneus	0.81023478	0.2883	0.1429	0.8571	-0.1297416
rh rostralanteriorcingulate	0.81023478	0.3933	0.1976	0.8024	-0.1199839
rh rostralmiddlefrontal	0.88354667	0.7675	0.6073	0.3927	0.05292762
rh superiorfrontal	0.81023478	0.2403	0.8821	0.1179	0.13951117
rh superiorparietal	0.81023478	0.5117	0.742	0.258	0.11258598
rh superiortemporal	0.88354667	0.7754	0.603	0.397	0.04736631
rh supramarginal	0.81023478	0.3573	0.8218	0.1782	0.11287695
rh frontalpole	0.81023478	0.2554	0.8739	0.1261	0.10929698
rh temporalpole	0.96133731	0.9472	0.4713	0.5287	-0.0083788
rh transversetemporal	0.81023478	0.1393	0.0697	0.9303	-0.1483127
rh insula	0.81023478	0.3896	0.1962	0.8038	-0.0954032

Network-based statistics, non-parametric permutation two-tailed t-tests FDR correction at a P<0.05

FDR, false discovery rate; lh, left hemisphere; PreHD, premanifest Huntington’s disease; rh, right hemisphere

Table S9 Permutation analysis of nodal strength in late preHD

Regions	P_{FDR}	$P_{uncorrected}$	late preHD < controls	late preHD > controls	Difference
lh bankssts	0.98194615	0.6639	0.6735	0.3265	0.07575199
lh caudalanteriorcingulate	0.92344	0.4742	0.2412	0.7588	-0.0568978
lh caudalmiddlefrontal	0.99269851	0.9781	0.5056	0.4944	0.00267388
lh cuneus	0.55954286	0.0576	0.9735	0.0265	0.25736049
lh entorhinal	0.98194615	0.6636	0.6722	0.3278	0.06010339
lh fusiform	0.92344	0.4395	0.7884	0.2116	0.08892077
lh inferiorparietal	0.92344	0.4371	0.224	0.776	-0.0676635
lh inferiortemporal	0.99028923	0.9385	0.5381	0.4619	0.00909166
lh isthmuscingulate	0.37264	0.0274	0.0129	0.9871	-0.2385326
lh lateraloccipital	0.98194615	0.5918	0.2986	0.7014	-0.0745951
lh lateralorbitofrontal	0.99028923	0.9438	0.5235	0.4765	0.00667825
lh lingual	0.92344	0.3294	0.8364	0.1636	0.12028766
lh medialorbitofrontal	0.92344	0.4413	0.2231	0.7769	-0.0819821
lh middletemporal	0.92344	0.395	0.8032	0.1968	0.09832199
lh parahippocampal	0.1972	0.0079	0.9959	0.0041	0.25740856
lh paracentral	0.1972	0.0087	0.0049	0.9951	-0.2754825
lh parsopercularis	0.99028923	0.8311	0.4245	0.5755	-0.0234057
lh parsorbitalis	0.99028923	0.9115	0.5505	0.4495	0.00914857
lh parstriangularis	0.92344	0.4402	0.223	0.777	-0.0703169
lh pericalcarine	0.92344	0.3539	0.8227	0.1773	0.11203386
lh postcentral	0.92344	0.3905	0.193	0.807	-0.1101495
lh posteriorcingulate	0.92344	0.2203	0.113	0.887	-0.1313375
lh precentral	0.92344	0.463	0.2266	0.7734	-0.0750616
lh precuneus	0.98194615	0.6562	0.3243	0.6757	-0.0405602
lh rostralanteriorcingulate	0.99028923	0.8882	0.5676	0.4324	0.01509353
lh rostralmiddlefrontal	0.99269851	0.975	0.505	0.495	0.00390876
lh superiorfrontal	0.92344	0.3278	0.1627	0.8373	-0.0909005
lh superiorparietal	0.55954286	0.051	0.0258	0.9742	-0.2345447
lh superiortemporal	0.9999	0.9999	0.5054	0.4946	-1.78E-05
lh supramarginal	0.99028923	0.8979	0.5491	0.4509	0.01380769
lh frontalpole	0.99028923	0.8175	0.596	0.404	0.01926172
lh temporalpole	0.99028923	0.8896	0.4414	0.5586	-0.0128866
lh transversetemporal	0.92344	0.2992	0.1494	0.8506	-0.0875444
lh insula	0.3196	0.0188	0.0099	0.9901	-0.1925337
rh bankssts	0.98194615	0.7509	0.3792	0.6208	-0.0475393
rh caudalanteriorcingulate	0.98194615	0.6065	0.2983	0.7017	-0.0441179
rh caudalmiddlefrontal	0.98194615	0.5271	0.2672	0.7328	-0.0590386
rh cuneus	0.98194615	0.701	0.6483	0.3517	0.0488741
rh entorhinal	0.92344	0.1826	0.9152	0.0848	0.18349858
rh fusiform	0.92344	0.3826	0.8131	0.1869	0.10631137
rh inferiorparietal	0.99028923	0.8181	0.5994	0.4006	0.02000184
rh inferiortemporal	0.92344	0.4458	0.7811	0.2189	0.09339294

rh isthmuscingulate	0.92344	0.1885	0.0954	0.9046	-0.1425003
rh lateraloccipital	0.99028923	0.9407	0.4689	0.5311	-0.0098525
rh lateralorbitofrontal	0.99028923	0.9466	0.4665	0.5335	-0.0074659
rh lingual	0.92344	0.3886	0.8074	0.1926	0.09595967
rh medialorbitofrontal	0.98194615	0.7339	0.6346	0.3654	0.03254435
rh middletemporal	0.98194615	0.7275	0.6395	0.3605	0.03675657
rh parahippocampal	0.1156	0.0017	0.9992	0.0008	0.27940362
rh paracentral	0.78115	0.0919	0.0453	0.9547	-0.1819034
rh parsopercularis	0.98194615	0.6761	0.3466	0.6534	-0.0461335
rh parsorbitalis	0.99028923	0.7924	0.393	0.607	-0.0204526
rh parstriangularis	0.99028923	0.928	0.5449	0.4551	0.00907847
rh pericalcarine	0.98194615	0.7476	0.3798	0.6202	-0.0482356
rh postcentral	0.98194615	0.7043	0.3592	0.6408	-0.0534361
rh posteriorcingulate	0.8908	0.1179	0.0586	0.9414	-0.1773021
rh precentral	0.92344	0.3525	0.1796	0.8204	-0.0837866
rh precuneus	0.92344	0.3141	0.1604	0.8396	-0.1055914
rh rostralanteriorcingulate	0.98194615	0.6115	0.6999	0.3001	0.05192898
rh rostralmiddlefrontal	0.92344	0.4018	0.8015	0.1985	0.10562784
rh superiorfrontal	0.98194615	0.696	0.3428	0.6572	-0.0359562
rh superiorparietal	0.92344	0.2678	0.1341	0.8659	-0.1378031
rh superiortemporal	0.92344	0.4363	0.224	0.776	-0.0938948
rh supramarginal	0.92344	0.2779	0.1374	0.8626	-0.0945013
rh frontalpole	0.98194615	0.6993	0.35	0.65	-0.0258142
rh temporalpole	0.92344	0.4753	0.2338	0.7662	-0.06861
rh transversetemporal	0.98194615	0.6958	0.3523	0.6477	-0.0309919
rh insula	0.92344	0.3911	0.1963	0.8037	-0.0738569

Network-based statistics, non-parametric permutation two-tailed t-tests FDR correction at a $P<0.05$

FDR, false discovery rate; lh, left hemisphere; PreHD, premanifest Huntington's disease; rh, right hemisphere

Table S10 Permutation analysis of nodal strength in mHD

Regions	P_{FDR}	$P_{uncorrected}$	mHD<controls	mHD>controls	Difference
lh bankssts	0.08120769	0.0611	0.0312	0.9688	-0.3107188
lh caudalanteriorcingulate	0.00174857	0.0009	0.0006	0.9994	-0.266263
lh caudalmiddlefrontal	0.00090667	0.0004	0.0003	0.9997	-0.3226324
lh cuneus	0.31737049	0.283	0.1405	0.8595	-0.1240602
lh entorhinal	0.9621	0.9621	0.5164	0.4836	0.00539199
lh fusiform	0.00523077	0.003	0.0013	0.9987	-0.3381476
lh inferiorparietal	0.00030909	0.0001	1.00E-04	0.9999	-0.4278654
lh inferiortemporal	0.00030909	0.0001	1.00E-04	0.9999	-0.4964163
lh isthmuscingulate	0.0153	0.0099	0.0058	0.9942	-0.2530831
lh lateraloccipital	0.00030909	0.0001	1.00E-04	0.9999	-0.6802616
lh lateralorbitofrontal	0.00174857	0.0009	0.0005	0.9995	-0.3050446
lh lingual	0.03555833	0.0251	0.0128	0.9872	-0.2463653
lh medialorbitofrontal	0.08120769	0.0621	0.0306	0.9694	-0.1667199
lh middletemporal	0.00207778	0.0011	0.0008	0.9992	-0.3359727
lh parahippocampal	0.03110638	0.0215	0.0119	0.9881	-0.1958004
lh paracentral	0.00052308	0.0002	1.00E-04	0.9999	-0.3674445
lh parsopercularis	0.00030909	0.0001	1.00E-04	0.9999	-0.4499887
lh parsorbitalis	0.0153	0.0096	0.0048	0.9952	-0.208667
lh parstriangularis	0.00294054	0.0016	0.0009	0.9991	-0.2672177
lh pericalcarine	0.92078182	0.8937	0.5533	0.4467	0.01392868
lh postcentral	0.0306	0.0207	0.0108	0.9892	-0.261441
lh posteriorcingulate	0.22405424	0.1944	0.0948	0.9052	-0.1313512
lh precentral	0.0010625	0.0005	0.0002	0.9998	-0.3182818
lh precuneus	0.00030909	0.0001	1.00E-04	0.9999	-0.4794591
lh rostralanteriorcingulate	0.00030909	0.0001	1.00E-04	0.9999	-0.5085288
lh rostralmiddlefrontal	0.12616429	0.1039	0.0524	0.9476	-0.1736318
lh superiorfrontal	0.00075556	0.0003	0.0002	0.9998	-0.3432734
lh superiorparietal	0.00030909	0.0001	1.00E-04	0.9999	-0.7457861
lh superiortemporal	0.20388276	0.1739	0.0879	0.9121	-0.1318653
lh supramarginal	0.00030909	0.0001	1.00E-04	0.9999	-0.5376195
lh frontalpole	0.01783111	0.0118	0.0056	0.9944	-0.1591294
lh temporalpole	0.31894194	0.2908	0.1495	0.8505	-0.0752134
lh transversetemporal	0.00090667	0.0004	0.0002	0.9998	-0.256363
lh insula	0.00030909	0.0001	1.00E-04	0.9999	-0.5919265
rh bankssts	0.07507755	0.0541	0.0262	0.9738	-0.2710274
rh caudalanteriorcingulate	0.00090667	0.0004	0.0003	0.9997	-0.2741694
rh caudalmiddlefrontal	0.00052308	0.0002	1.00E-04	0.9999	-0.3885874
rh cuneus	0.12717193	0.1066	0.0537	0.9463	-0.1740318
rh entorhinal	0.81934769	0.7832	0.3917	0.6083	-0.0312668
rh fusiform	0.00123636	0.0006	0.0004	0.9996	-0.3592655
rh inferiorparietal	0.00030909	0.0001	1.00E-04	0.9999	-0.375167
rh inferiortemporal	0.00030909	0.0001	1.00E-04	0.9999	-0.4920965
rh isthmuscingulate	0.0153	0.0099	0.0052	0.9948	-0.2586996

rh lateraloccipital	0.00030909	0.0001	1.00E-04	0.9999	-0.9042307
rh lateralorbitofrontal	0.08249811	0.0643	0.0346	0.9654	-0.1829836
rh lingual	0.01177561	0.0071	0.0035	0.9965	-0.2599036
rh medialorbitofrontal	0.00030909	0.0001	1.00E-04	0.9999	-0.390851
rh middletemporal	0.00030909	0.0001	1.00E-04	0.9999	-0.4566499
rh parahippocampal	0.07888	0.058	0.0284	0.9716	-0.1688174
rh paracentral	0.00030909	0.0001	1.00E-04	0.9999	-0.3847663
rh parsopercularis	0.00030909	0.0001	1.00E-04	0.9999	-0.4200292
rh parsorbitalis	0.0034	0.0019	0.001	0.999	-0.2582578
rh parstriangularis	0.08512593	0.0676	0.0358	0.9642	-0.1788434
rh pericalcarine	0.73365625	0.6905	0.6515	0.3485	0.04850073
rh postcentral	0.0010625	0.0005	0.0003	0.9997	-0.4199838
rh posteriorcingulate	0.12616429	0.103	0.0501	0.9499	-0.1462614
rh precentral	0.00052308	0.0002	0.0002	0.9998	-0.3379803
rh precuneus	0.00030909	0.0001	1.00E-04	0.9999	-0.3619325
rh rostralanteriorcingulate	0.00030909	0.0001	1.00E-04	0.9999	-0.5500951
rh rostralmiddlefrontal	0.94103881	0.9272	0.545	0.455	0.00973644
rh superiorfrontal	0.00030909	0.0001	1.00E-04	0.9999	-0.4431436
rh superiorparietal	0.00030909	0.0001	1.00E-04	0.9999	-0.6248941
rh superiortemporal	0.4884127	0.4525	0.2325	0.7675	-0.0901304
rh supramarginal	0.00030909	0.0001	1.00E-04	0.9999	-0.5401209
rh frontalpole	0.00986	0.0058	0.0027	0.9973	-0.171357
rh temporalpole	0.31737049	0.2847	0.1445	0.8555	-0.0846532
rh transversetemporal	0.00052308	0.0002	0.0002	0.9998	-0.2940753
rh insula	0.00030909	0.0001	1.00E-04	0.9999	-0.404296

Network-based statistics, non-parametric permutation two-tailed t-tests FDR correction at a $P<0.05$

FDR, false discovery rate; mHD, manifest Huntington’s disease

Table S11 Correlation between plasma NfL and nodal strength

	Early preHD			Late preHD			Manifest HD		
	rho	Pval	P_FDR	rho	Pval	P_FDR	rho	Pval	P_FDR
lh_bankssts	-0.01	0.930	0.989	-0.09	0.399	0.744	-0.20	0.083	0.137
lh_caudalanteriorcingulate	-0.04	0.782	0.989	-0.01	0.939	1.000	-0.13	0.256	0.334
lh_caudalmiddlefrontal	0.12	0.378	0.989	0.04	0.727	0.932	-0.23	0.038	0.083
lh_cuneus	0.18	0.187	0.989	-0.21	0.038	0.325	-0.26	0.021	0.057
lh_entorhinal	-0.02	0.866	0.989	0.00	0.990	1.000	-0.22	0.053	0.105
lh_fusiform	-0.11	0.424	0.989	0.09	0.389	0.744	-0.28	0.012	0.040
lh_inferiorparietal	0.02	0.910	0.989	-0.13	0.215	0.544	-0.23	0.042	0.086
lh_inferiortemporal	-0.02	0.899	0.989	-0.03	0.750	0.932	-0.27	0.015	0.044
lh_isthmuscingulate	-0.01	0.922	0.989	0.01	0.893	1.000	-0.29	0.008	0.040
lh_lateraloccipital	-0.01	0.968	0.989	-0.19	0.063	0.325	-0.37	0.001	0.015
lh_lateralorbitofrontal	0.06	0.674	0.989	-0.09	0.392	0.744	-0.28	0.013	0.042
lh_lingual	0.07	0.615	0.989	-0.16	0.112	0.422	-0.02	0.885	0.926
lh_medialorbitofrontal	0.09	0.527	0.989	-0.12	0.258	0.584	-0.20	0.070	0.118
lh_middletemporal	-0.12	0.406	0.989	-0.16	0.126	0.430	-0.27	0.015	0.044
lh_parahippocampal	0.00	0.976	0.989	-0.13	0.205	0.544	-0.37	0.001	0.015
lh_paracentral	0.16	0.251	0.989	-0.02	0.841	1.000	-0.34	0.002	0.020
lh_parsopercularis	0.06	0.652	0.989	0.08	0.440	0.767	-0.21	0.067	0.118
lh_parsorbitalis	0.18	0.189	0.989	-0.07	0.468	0.795	-0.10	0.377	0.442
lh_parstriangularis	0.03	0.838	0.989	-0.18	0.071	0.325	-0.16	0.158	0.225
lh_pericalcarine	0.13	0.340	0.989	-0.03	0.785	0.954	0.00	0.996	0.996
lh_postcentral	0.06	0.679	0.989	-0.04	0.719	0.932	0.00	0.980	0.995
lh_posteriorcingulate	0.05	0.698	0.989	-0.11	0.280	0.613	-0.09	0.414	0.462
lh_precentral	-0.05	0.711	0.989	-0.03	0.742	0.932	-0.28	0.011	0.040
lh_precuneus	0.10	0.464	0.989	-0.09	0.405	0.744	-0.30	0.006	0.040
lh_rostralanteriorcingulate	0.19	0.174	0.989	-0.23	0.021	0.325	-0.22	0.054	0.105
lh_rostralmiddlefrontal	0.07	0.627	0.989	-0.25	0.013	0.325	-0.25	0.026	0.067
lh_superiorfrontal	0.07	0.609	0.989	-0.13	0.216	0.544	-0.28	0.011	0.040
lh_superiorparietal	-0.01	0.925	0.989	-0.15	0.142	0.460	-0.21	0.068	0.118
lh_superiortemporal	0.01	0.942	0.989	-0.07	0.492	0.816	-0.16	0.159	0.225
lh_supramarginal	-0.04	0.790	0.989	-0.19	0.058	0.325	-0.27	0.017	0.048

lh_frontalpole	-0.17	0.231	0.989	-0.16	0.116	0.422	-0.34	0.002	0.020
lh_temporalpole	0.01	0.953	0.989	-0.01	0.948	1.000	-0.09	0.412	0.462
lh_transversetemporal	0.00	0.984	0.989	0.01	0.928	1.000	-0.09	0.405	0.462
lh_insula	-0.15	0.268	0.989	-0.04	0.710	0.932	-0.11	0.325	0.400
rh_bankssts	-0.04	0.775	0.989	0.05	0.637	0.921	-0.05	0.645	0.685
rh_caudalanteriorcingulate	0.03	0.827	0.989	-0.06	0.538	0.836	-0.11	0.329	0.400
rh_caudalmiddlefrontal	0.03	0.814	0.989	-0.20	0.047	0.325	-0.34	0.002	0.020
rh_cuneus	0.07	0.621	0.989	-0.20	0.051	0.325	-0.09	0.421	0.462
rh_entorhinal	0.16	0.246	0.989	0.08	0.430	0.767	-0.06	0.617	0.666
rh_fusiform	-0.02	0.899	0.989	0.00	0.976	1.000	-0.20	0.069	0.118
rh_inferiorparietal	-0.12	0.371	0.989	-0.19	0.061	0.325	-0.28	0.011	0.040
rh_inferiortemporal	0.06	0.659	0.989	-0.01	0.942	1.000	-0.39	0.000	0.015
rh_isthmuscingulate	0.07	0.629	0.989	0.01	0.917	1.000	-0.15	0.172	0.238
rh_lateraloccipital	0.13	0.348	0.989	-0.30	0.002	0.162	-0.23	0.039	0.083
rh_lateralorbitofrontal	0.06	0.665	0.989	-0.12	0.231	0.559	-0.23	0.037	0.083
rh_lingual	-0.11	0.430	0.989	-0.22	0.028	0.325	-0.19	0.097	0.154
rh_medialorbitofrontal	0.02	0.895	0.989	-0.13	0.196	0.544	-0.19	0.092	0.149
rh_middletemporal	0.03	0.848	0.989	-0.10	0.343	0.706	-0.33	0.003	0.025
rh_parahippocampal	0.10	0.491	0.989	-0.16	0.118	0.422	-0.30	0.008	0.040
rh_paracentral	0.00	0.978	0.989	0.04	0.714	0.932	-0.29	0.009	0.040
rh_parsopercularis	-0.15	0.293	0.989	-0.23	0.021	0.325	-0.10	0.377	0.442
rh_parsorbitalis	0.26	0.057	0.989	-0.05	0.615	0.908	-0.28	0.010	0.040
rh_parstriangularis	0.18	0.202	0.989	-0.07	0.517	0.836	-0.24	0.034	0.083
rh_pericalcarine	-0.10	0.470	0.989	-0.04	0.715	0.932	0.13	0.260	0.334
rh_postcentral	-0.16	0.242	0.989	-0.06	0.541	0.836	-0.18	0.119	0.175
rh_posteriorcingulate	0.04	0.771	0.989	0.01	0.888	1.000	-0.18	0.107	0.161
rh_precentral	-0.02	0.899	0.989	-0.17	0.086	0.367	-0.14	0.201	0.269
rh_precuneus	0.00	0.987	0.989	-0.18	0.072	0.325	-0.34	0.002	0.020
rh_rostralanteriorcingulate	-0.11	0.445	0.989	-0.13	0.191	0.544	-0.24	0.029	0.074
rh_rostralmiddlefrontal	0.16	0.239	0.989	-0.06	0.573	0.865	-0.19	0.100	0.155
rh_superiorfrontal	0.05	0.721	0.989	-0.18	0.069	0.325	-0.28	0.012	0.040
rh_superiorparietal	-0.20	0.140	0.989	-0.12	0.239	0.559	-0.31	0.005	0.038
rh_superiortemporal	0.07	0.600	0.989	0.01	0.958	1.000	-0.15	0.177	0.240
rh_supramarginal	0.04	0.762	0.989	-0.14	0.172	0.532	-0.28	0.011	0.040

rh_frontalpole	-0.01	0.953	0.989	-0.19	0.064	0.325	-0.01	0.908	0.936
rh_temporalpole	0.02	0.900	0.989	0.00	1.000	1.000	-0.12	0.280	0.353
rh_transversetemporal	0.07	0.611	0.989	0.03	0.754	0.932	-0.23	0.037	0.083
rh_insula	0.00	0.989	0.989	-0.10	0.322	0.684	-0.20	0.068	0.118

Network-based statistics, non-parametric permutation two-tailed t-tests FDR correction at a P<0.05

FDR, false discovery rate; lh, left hemisphere; NfL, plasma neurofilament light; rh, right hemisphere.

Table S12 Correlations in node strength between cohorts

Cohorts	Rho	P value	P_{FDR}
mHD – late preHD	0.382	0.001	0.04605
mHD – early preHD	0.007	0.952	0.9520
Late preHD-early preHD	0.262	0.0307	0.00300

Pearson's correlation, two-tailed, FDR correction at a P<0.05
FDR, false discovery rate; mHD, manifest HD; preHD, premanifest HD

Table S13 Correlations in connection strength between cohorts

Cohorts	Rho	P value	P_{FDR}
mHD – late preHD	0.386	$1.74 * 10^{-164}$	$5.11 * 10^{-164}$
mHD – early preHD	0.119	$6.16 * 10^{-16}$	$9.25 * 10^{-16}$
Late preHD-early preHD	0.235	$4.62 * 10^{-59}$	$6.93 * 10^{-59}$

Pearson's correlation, two-tailed, FDR correction at a $P<0.05$

mHD, manifest HD; preHD, premanifest HD

Table S14 Correlations between organisational principles and nodal strength

		rho			Pspin (FDR-corrected)		
		Early preHD	Late preHD	mHD	Early preHD	Late preHD	mHD
STRUCTURAL	Laminar similarity	0.11	0.38	0.00	0.555	0.086	0.956
	White matter connectivity	0.29	0.54	0.06	0.243	0.096	0.580
	Euclidean distance	0.23	0.61	0.00	0.349	0.051	0.966
FUNCTIONAL	FDG-PET	0.05	0.61	0.40	0.812	***0.011**	***0.007**
	rsfMRI	0.22	0.40	0.23	0.389	0.138	0.158
	Dynamic fMRI	0.24	0.38	0.28	0.419	0.097	0.148
	MEG	0.31	0.64	0.06	0.078	***0.024**	0.757
CELL AUTONOMOUS	Gene expression	0.33	0.58	0.42	0.128	***0.021**	***0.014**
	Receptor similarity	0.44	0.45	0.24	***0.016**	0.110	0.128

Spearman's correlation. Statistical significance was assessed through non parametric FDR-corrected spin permutation tests, two-tailed, DF = 66.

*FDR-corrected $P_{spin} < 0.05$

FDG-PET, [F^{18}]- fluordoxyglucose positron emission tomography; FDR, false discovery rate; HD, Huntington's disease; MEG, magnetoencephalography; preHD, premanifest Huntington's disease; rsfMRI, resting-state functional MRI.

Table S15 Receptome gradient correlations

	rho			Pspin			FDR-corrected Pspin		
	Early preHD	Late preHD	mHD	Early preHD	Late preHD	mHD	Early preHD	Late preHD	mHD
Gradient 1	-0.32*	-0.53*	-0.04	0.001	0.021	0.911	0.003	0.0315	0.911
Gradient 2	-0.02	-0.23	-0.11	0.898	0.44	0.711	0.898	0.898	0.898
Gradient 3	-0.14	0.07	0.03	0.286	0.725	0.868	0.858	0.868	0.868

* $P_{FDR} < 0.05$

Spearman rank correlations. Statistical significance was assessed through non parametric FDR-corrected spin permutation tests, two-tailed, DF = 98.

FDR, false-discovery rate; mHD, manifest Huntington's disease; preHD, premanifest Huntington's disease

Table S16 Dominance analysis between nodal strength and PET neurotransmitter distribution

mHD*			Late preHD*			Early preHD*		
Neurotransmitter	R	% to the model	Neurotransmitter	R	% to the model	Neurotransmitter	R	% to the model
5HT1a	0.027	7.3	5HT1a	0.166	26.7	5HT1a	0.010	2.8
5HT1b	0.003	0.8	5HT1b	0.049	7.9	5HT1b	0.011	3.0
5HT2a	-0.002	-0.5	5HT2a	0.001	0.1	5HT2a	0.007	2.0
5HT4	0.003	0.9	5HT4	0.002	0.3	5HT4	0.025	6.8
5HT6	-0.002	-0.6	5HT6	0.008	1.2	5HT6	0.005	1.2
5HTT	0.133	36.4	5HTT	0.011	1.8	5HTT	0.030	8.3
A4B2	0.022	5.9	A4B2	0.012	1.9	A4B2	0.029	8.0
CB1	0.004	1.2	CB1	0.007	1.2	CB1	0.034	9.3
D1	0.016	4.4	D1	0.007	1.2	D1	0.002	0.7
D2	0.025	6.8	D2	0.015	2.3	D2	0.000	0.1
DAT	0.007	1.9	DAT	0.057	9.1	DAT	0.017	4.6
GABAa	0.003	0.7	GABAa	0.007	1.2	GABAa	0.002	0.7
H3	0.048	13.1	H3	0.019	3.0	H3	0.017	4.6
M1	-0.004	-1.0	M1	0.005	0.8	M1	-0.003	-0.7
mGluR5	0.021	5.6	mGluR5	0.025	4.0	mGluR5	0.024	6.7
MOR	0.001	0.3	MOR	0.049	7.8	MOR	0.033	9.0
NET	0.018	5.0	NET	0.045	7.3	NET	0.018	5.0
NMDA	0.029	8.0	NMDA	0.007	1.1	NMDA	0.010	2.8
VAChT	0.014	3.8	VAChT	0.131	21.1	VAChT	0.091	25.1
Total fit	0.365	100.0	Total fit	0.623	100.0	Total fit	0.362	100.0

FDR-corrected spin permutation tests, one-sided, N = 68 regions

* FDR-Pspin <0.05

FDR, false-discovery rate; mHD, manifest Huntington's disease; preHD, premanifest Huntington's disease

Table S17 Dominance analysis between nodal strength in early preHD and autoradiography neurotransmitter distribution

SUPRAGRANULAR			GRANULAR*			INFRAGRANULAR*		
Neurotransmitter	R	% to the model	Neurotransmitter	R	% to the model	Neurotransmitter	R	% to the model
AMPA	0.09	24.4	AMPA	0.05	7.8	AMPA	0.06	11.4
NMDA	-0.01	-1.5	NMDA	0.04	6.1	NMDA	-0.01	-1.2
kainate	0.03	7.7	kainate	0.03	4.6	kainate	0.10	17.8
GABAa	0.09	23.3	GABAa	0.10	16.8	GABAa	0.15	26.6
GABAa/BZ	-0.01	-3.8	GABAa/BZ	0.01	1.4	GABAa/BZ	0.00	-0.8
GABAb	0.00	0.2	GABAb	0.01	2.3	GABAb	0.01	2.4
m1	0.01	1.5	m1	-0.01	-1.7	m1	-0.01	-1.3
m2	0.02	6.1	m2	0.08	13.0	m2	0.06	11.8
m3	-0.02	-6.4	m3	-0.01	-1.6	m3	-0.02	-3.0
a4b2	-0.01	-1.8	a4b2	0.04	6.6	a4b2	0.01	2.0
a1	-0.01	-3.8	a1	-0.02	-2.5	a1	0.00	-0.6
a2	-0.02	-4.1	a2	0.01	1.9	a2	0.02	3.3
5-HT1a	0.02	6.2	5-HT1a	0.03	4.2	5-HT1a	0.01	1.5
5-HT2	0.17	45.1	5-HT2	0.18	30.1	5-HT2	0.15	27.5
D1	0.03	7.0	D1	0.07	11.1	D1	0.01	2.5
Total fit	0.38	100	Total fit	0.61	100	Total fit	0.55	100

FDR-corrected spin permutation tests, one-sided, N = 68 regions

* FDR-Pspin <0.05

FDR, false-discovery rate; mHD, manifest Huntington's disease; preHD, premanifest Huntington's disease

Table S18 Dominance analysis between nodal strength in late preHD and autoradiography neurotransmitter distribution

SUPRAGRANULAR			GRANULAR			INFRAGRANULAR		
Neurotransmitter	R	% to the model	Neurotransmitter	R	% to the model	Neurotransmitter	R	% to the model
AMPA	0.02	5.0	AMPA	0.01	1.7	AMPA	0.00	1.3
NMDA	0.18	43.9	NMDA	0.12	29.5	NMDA	0.16	53.0
kainate	-0.01	-3.4	kainate	0.01	1.7	kainate	0.00	-1.1
GABAa	0.01	1.7	GABAa	0.03	7.3	GABAa	-0.01	-2.4
GABAa/BZ	0.07	17.6	GABAa/BZ	0.06	13.0	GABAa/BZ	0.14	46.1
GABAb	0.01	2.7	GABAb	0.02	5.4	GABAb	0.02	6.6
m1	0.00	0.1	m1	-0.01	-1.3	m1	-0.01	-4.9
m2	0.00	-0.6	m2	-0.01	-2.1	m2	-0.02	-7.2
m3	0.00	0.3	m3	0.00	0.5	m3	0.01	2.8
a4b2	0.00	-0.9	a4b2	-0.01	-2.0	a4b2	-0.02	-5.9
a1	-0.01	-2.8	a1	-0.01	-2.2	a1	0.01	2.4
a2	0.00	-0.2	a2	0.07	17.2	a2	0.03	9.8
5-HT1a	0.08	18.4	5-HT1a	0.03	6.0	5-HT1a	-0.01	-3.5
5-HT2	0.03	6.5	5-HT2	0.06	14.1	5-HT2	-0.01	-3.7
D1	0.05	11.7	D1	0.05	11.2	D1	0.02	6.7
Total fit	0.42	100.0	Total fit	0.42	100.0	Total fit	0.30	100.0

No significant results

FDR-corrected spin permutation tests, one-sided, N = 68 regions

FDR, false-discovery rate; mHD, manifest Huntington's disease; preHD, premanifest Huntington's disease

Table S19 Dominance analysis between nodal strength in mHD and autoradiography neurotransmitter distribution

SUPRAGRANULAR			GRANULAR			INFRAGRANULAR		
Neurotransmitter	R	% to the model	Neurotransmitter	R	% to the model	Neurotransmitter	R	% to the model
AMPA	0.05	-34.21	AMPA	-0.01	-6.9	AMPA	0.74	72.73
NMDA	-0.01	6.98	NMDA	0.08	96.8	NMDA	0.02	2.31
kainate	-0.03	22.50	kainate	-0.03	-30.4	kainate	-0.03	-3.40
GABAa	-0.02	17.86	GABAa	0.08	89.0	GABAa	0.10	10.14
GABAa/BZ	-0.01	5.37	GABAa/BZ	-0.01	-11.8	GABAa/BZ	0.11	10.87
GABAb	-0.02	11.71	GABAb	-0.02	-19.9	GABAb	-0.02	-2.34
m1	-0.02	16.29	m1	0.00	4.0	m1	-0.02	-2.20
m2	0.05	-35.49	m2	0.03	40.5	m2	0.07	6.42
m3	-0.04	25.34	m3	-0.03	-36.7	m3	-0.01	-0.90
a4b2	-0.04	25.38	a4b2	-0.01	-10.1	a4b2	-0.03	-2.84
a1	-0.03	23.39	a1	-0.02	-22.3	a1	-0.02	-1.60
a2	-0.02	11.32	a2	-0.01	-17.1	a2	0.00	-0.46
5-HT1a	-0.02	13.59	5-HT1a	-0.02	-18.9	5-HT1a	-0.01	-1.23
5-HT2	0.01	-6.33	5-HT2	0.04	48.6	5-HT2	0.09	8.37
D1	0.01	-3.70	D1	0.00	-4.7	D1	0.04	4.12
Total fit	-0.14	100.00	Total fit	0.08	100	Total fit	1.02	100

No significant results
FDR-corrected spin permutation tests, one-sided, N = 68 regions
FDR, false-discovery rate; mHD, manifest Huntington's disease

Table S20 Demographics from late preHD participants from TrackHD and TrackOn HD

	Late preHD		
	Track HD	TrackOn HD	P value
N	103	85	N/A
Age	40.76 (8.40)	43.66 (8.92)	P = 0.8138
Sex (M:F)	47:53	39:46	P = 0.8793
Site	Leiden: 28<br>London: 24<br>Paris: 27<br>Vancouver: 24	Leiden: 18<br>London: 20<br>Paris: 25<br>Vancouver: 22	P = 0.8044
DBS	292.66 (47.71)	304.31 (55.29)	P = 0.8728
CAG	43.08 (2.31)	42.84 (2.33)	P = 0.9423

Mean (SD)

DBS, disease burden score; F, Female; M, male; SD, standard deviation

Table S21 Methodological details for each PET tracer

target	tracer	scanner	N	n_male	n_female	age_years	sd_age	parameter	modelling_method	reference_region	scan_length_min	modeling_notes	primary_reference	see_also	contact
5HT1a	[11C]CUMI-101	HRRT	8	3	5	28.4	8.8	Bmax	MRTM2	cerebellum	120	originally BPnd, converted to Bmax using autoradiography densities	beliveau2017jneurosci		
5HT1a	[*carbonyl*-11C]WAY-100635	GE_Advance	35	18	17	26.3	5.2	BPnd	MRTM2	cerebellum	90	k2' fixed to insula	savli2012neuroimage	fink2009neuroimage	
5HT1b	[11C]AZ10419369	HRRT	36	24	12	27.8	6.9	Bmax	MRTM2	cerebellum	90	originally BPnd, converted to Bmax using autoradiography densities	beliveau2017jneurosci		
5HT1b	[11C]P943	HRRT	65	49	16	33.73	9.73	BPnd	MRTM2	cerebellum	120	t* = 20 min; k2' fixed to median k2' all voxels with BP>0.5 in first-stage MRTM analysis	gallezot2010jcerebbloodflowmetab	Murrough et al. 2010 Psychopharm PMID20480149; Murrough et al. 2011 Arch Gen Psychiatry PMID21893657; Matuskey et al. 2014 Biol Psychiatry PMID24433854; Pittenger et al 2016 J Affect Disord PMID26919057; Saricicek et al 2015 Eur J Nucl Med Mol Imaging PMID25427881; Baldassarri et al	kelly.smart@yale.edu; richard.carson@yale.edu

														2020 Synapse PMID32324935	
5HT1b	[11C]P943	HRRT	23	15	8	28.7	7	BPnd	MRTM2	cerebellum	120	k2' fixed to calcarine fissure	savli2012neuroimage		
5HT2a	[18F]ALTANSERIN	HR+	19	11	8	28.2	5.7	BPnd		cerebellum	120-180	BPnd = (C_voxel - C_cerebellum)/C_parent-compound, C = plasma radioactivity concentration	savli2012neuroimage	hurlemann2018psychopharmacology	
5HT2a	[11C]CIMBI-36	HRRT	29	15	14	22.6	2.7	Bmax	MRTM2	cerebellum	120	originally BPnd, converted to Bmax using autoradiography densities	beliveau2017jneurosci		
5HT2a	[11C]MDL100907	HR+	3	3	0	41.14	9.94	BPnd	SRTM	cerebellum	120	parametric image validated by comparison to ROI analysis. PFC unreliable.	talbot2012neuroimage	hinz2007jcerebbloodflowmetab	kelly.smart@yale.edu; richard.carson@yale.edu
5HT4	[11C]SB207145	HRRT	59	41	18	25.9	5.3	Bmax	MRTM2	cerebellum	120	originally BPnd, converted to Bmax using autoradiography densities	beliveau2017jneurosci		
5HT6	[11C]GSK215083	HR+	30	30	0	36.6	9.04	BPnd	MA1	cerebellum	120	t* = 30 min; BPnd = (Vt_voxel - Vt_cerebellum)/Vt_cerebellum; Modelling	radhakrishnan2018jnuclmed	Radhakrishnan et al 2020 Psychiatry Res	kelly.smart@yale.edu; richard.carson@yale.edu

												method reference: parker2015jnuclmed (https://jnm.snmjournals.org/content/56/12/1901.long)		Neuroimaging PMID31760336	
5HTT	[11C]DASB	GE_Advance	18	12	6	30.5	9.5	BPnd	MRTM2	cerebellum	120	k2' fixed to thalamus	savli2012neuroimage		
5HTT	[11C]DASB	HRRT	100	29	71	25.1	5.8	Bmax	MRTM2	cerebellum	90	originally BPnd, converted to Bmax using autoradiography densities; exclude medium raphe in analyses	beliveau2017jneurosci		
a4b2	[18F]FLUBATINE	HRRT	30	20	10	33.50	10.71	Vt	equilibrium_analysis	NA	120	Bolus + infusion protocol with equilibrium period 90-120 min; exclude thalamus in analyses	hillmer2016neuroimage	Baldassarri et al 2017 Nicotine Tob Res PMID28460123	kelly.smart@yale.edu; richard.carson@yale.edu
CB1	[18F]FMPEP-D2	HRRT	22	11	11	27.5	8.05	Vt	Logan_graphical_method	NA	60		laurikainen2019neuroimage		jahi@utu.fi
CB1	[11C]OMAR	HRRT	77	49	28	30.01	8.87	Vt	MA1	NA	120	t* = 20 min	normandin2015jcerebbloodflowmetab	D'Souza et al 2016 Biol Psychiatry Cogn Neurosci Neuroimaging PMID26858993; Ranganathan et al 2016 Biol Psychiatry PMID26432420; Neumeister et al 2012 Alcohol Clin Exp Res PMID22551199	kelly.smart@yale.edu; richard.carson@yale.edu
D1	[11C]SCH23390	Biograph_mMR	13	6	7	33	13	BPnd	MRTM2	cerebellum	90		kaller2017eurjnuclmedmolimaging		
D2	[18F]FALLYPRIDE	HRRT	49	16	33	18.41	0.57	BPnd	SRTM	cerebellum	90		jaworska2020neuropsychopharmacology		sylvia.cox@mcgill.ca

D2	[11C]FLB457	HRRT	37	17	20	48.36	16.93	BPnd	SRTM	cerebellum	120		smith2017jcerebbloodflowmetab	Sandiego et al 2015 J Cereb Blood Flow Metab PMID25564239	kelly.smart@yale.edu; richard.carson@yale.edu
D2	[11C]FLB457	HR+	55	26	29	32.45	9.69	BPnd	SRTM	cerebellum	90		sandiego2015jcerebbloodflowmetab	Smith et al 2017 J Cereb Blood Flow Metab PMID29090626; Zakiniaeiz et al 2019 Neuropsychopharm PMID31269510; Slifstein et al 2015 JAMA Psychiatry PMID25651194; Sandiego et al 2018 Neuropsychopharm PMID28944773	kelly.smart@yale.edu; richard.carson@yale.edu
D2	[11C]RACLOPRIDE	HRRT	7	7	0	24	2	BPnd	SRTM	cerebellum	80		alakurtti2015jcerebbloodflowmetab		
DAT	[18F]FE-PE2I	mCT	6	6	0	31.06	7.7	BPnd	SRTM	cerebeluum	90	parametric image validated by comparison to ROI analysis	sasaki2012jnuclmed		kelly.smart@yale.edu; richard.carson@yale.edu
DAT	[123I]FP-CIT	(SPECT)	174	109	65	61	11	SUVR	NA	occipital_cortex		from PPMI, www.ppmi-info.org/	dukart2018scirep		
GABAa	[11C]FLUMAZENIL	HR+	6	6	0	43	4	Vt	RPM	NA	90		dukart2018scirep	myers2012jcerebbloodflowmetab	
GABAabz	[11C]FLUMAZENIL	HRRT	16	7	9	26.6	8	Bmax	Logan_analysis	NA	90	t* = 35 min; originally Vt, converted to Bmax using autoradiography densities	norgaard2021neuroimage		

H3	[11C]GSK189254	HRRT	8	7	1	31.69	8.95	Vt	MA1	NA	120	t* = 30 min; parametric image validated by comparison to ROI analysis	gallezot2017jcerebbloodflowmetab	ashworth2010jnuclmed	kelly.smart@yale.edu; richard.carson@yale.edu
M1	[11C]LSN3172176	HRRT	24	13	11	40.45	11.71	BPnd	SRTM2	cerebellum	120	k2' fixed to median k2' all voxels with BP>0.5 in first-stage SRTM analysis	naganawa2020jnuclmed		kelly.smart@yale.edu; richard.carson@yale.edu
NMDAR	[18F]GE-179	Biograph_mMR	10	6	4	40.6	13.3	Vt	Logan_analysis	NA	70	Use of an image-derived input function with venous sampling	galovic2021preprint		marian.galovic@usz.ch
NMDAR	[18F]GE-179	GE_Discovery 690 TOF	10	9	1	40.1	9.4	Vt	Logan_analysis	NA	90	Arterial input function	galovic2021neuroimage		marian.galovic@usz.ch, jpc44@cam.ac.uk
NMDAR	[18F]GE-179	Siemens/CTI ECAT EXACT HR+ model 962	9	6	3	42.2	16.2	Vt	Logan_analysis	NA	90	Arterial input function	mcginnity2014JNM	McGinnity et al 2015 JNNP PMID: 25991402	marian.galovic@usz.ch, colm.mcginnity@kcl.ac.uk
mGluR5	[11C]ABP688	HRRT	22	12	10	67.9	9.6	BPnd	SRTM	cerebellar_cortex	60		PI: Pedro Rosa-Neto		pedro.rosa@mcgill.ca
mGluR5	[11C]ABP688	HRRT	28	15	13	33.1	11.2	BPnd	SRTM	cerebellar_cortex	60		dubois2016eurjnuclmedmolimaging		eliane.kobayashi@mcgill.ca
mGluR5	[11C]ABP688	HRRT	73	25	48	19.9	3.04	BPnd	SRTM	cerebellar_cortex	60		smart2019eurjnuclmedmolimaging		sylvia.cox@mcgill.ca
MOR	[11C]CARFENTANIL	HRRT	39	19	20	39.38	5.05	BPnd	SRTM	occipital_cortex	69		turtonen2021biolpsychiatrycnni		jahi@utu.fi
MOR	[11C]CARFENTANIL	variable	204	132	72	32.3	10.8	BPnd	SRTM	occipital_cortex	variable	See Supplementary Information from Kantonen et al., 2020 for variable image protocols.	kantonen2020neuroimage		

NET	[11C]MRB	HR+	10			33.3		BPnd	MRTM2	occipital_cortex	120		hesse2017eurjnuclmedmolimaging		
NET	[11C]MRB	HRRT	77	50	27	33.4	9.17	BPnd	MRTM2	occipital_cortex	120	t* = 20 min; k2' fixed to 0.02096 (population value)	ding2010synapse	Li et al 2014 NeuroImage PMID24121204; Sanchez-Rangel et al 2020 J Int Obesity PMID31636373; Belfort-DeAguiar et al 2018 J Clin Endocrinol Metab PMID29590401	kelly.smart@yale.edu; richard.carson@yale.edu
VAChT	[18F]FEOBV	HRRT	3	0	3	66.6	0.94	SUVR	NA	white_matter	30		PI: Taylor W. Schmitz & R. Nathan Spreng (with Prevent AD)		tschmitz@uwo.ca; nathan.spreng@mcgill.ca
VAChT	[18F]FEOBV	Biograph_mMR	4	3	1	37	10.2	SUVR	NA	white_matter	180-210		PI: Lauri Tuominen & Synthia Guimond		lauri.tuominen@theroyal.ca; synthia.guimond@theroyal.ca
VAChT	[18F]FEOBV	GE_Discovery	5	4	1	68.3	3.1	SUVR	NA	white_matter	180		bedard2019sleepmed		bedard.marc-andre@uqam.ca
VAChT	[18F]FEOBV	HRRT	18	5	13	66.8	6.8	SUVR	NA	white_matter	180		aghourian2017molpsychiatry		bedard.marc-andre@uqam.ca

			Region	Pspin	FDR-corrected Pspin
cortex	functional connectivity	early preHD	lh_bankssts	0.021	0.283
			lh_caudalanteriorcingulate	0.342	0.478
			lh_caudalmiddlefrontal	0.090	0.358
			lh_cuneus	0.038	0.283
			lh_entorhinal	0.195	0.389
			lh_frontalpole	0.137	0.369
			lh_fusiform	0.027	0.283
			lh_inferiorparietal	0.180	0.383
			lh_inferiortemporal	0.027	0.283
			lh_insula	0.420	0.485
			lh_isthmuscingulate	0.285	0.478
			lh_lateraloccipital	0.007	0.215
			lh_lateralorbitofrontal	0.276	0.478
			lh_lingual	0.180	0.383
			lh_medialorbitofrontal	0.372	0.478
			lh_middletemporal	0.106	0.367
			lh_paracentral	0.476	0.487
			lh_parahippocampal	0.174	0.383
			lh_parsopercularis	0.454	0.487
			lh_parsorbitalis	0.171	0.383
			lh_parstriangularis	0.129	0.369
			lh_pericalcarine	0.010	0.215
			lh_postcentral	0.422	0.485
			lh_posteriorcingulate	0.463	0.487
			lh_precentral	0.400	0.478
			lh_precuneus	0.454	0.487
			lh_rostralanteriorcingulate	0.336	0.478
			lh_rostralmiddlefrontal	0.297	0.478
			lh_superiorfrontal	0.152	0.382
			lh_superiorparietal	0.074	0.347
			lh_superiortemporal	0.365	0.478
			lh_supramarginal	0.364	0.478
			lh_temporalpole	0.069	0.347
			lh_transversetemporal	0.368	0.478
			rh_bankssts	0.108	0.367
			rh_caudalanteriorcingulate	0.324	0.478
			rh_caudalmiddlefrontal	0.219	0.413
			rh_cuneus	0.044	0.296
			rh_entorhinal	0.122	0.369
			rh_frontalpole	0.189	0.388
			rh_fusiform	0.009	0.215
			rh_inferiorparietal	0.203	0.394
			rh_inferiortemporal	0.077	0.347
			rh_insula	0.485	0.487

			rh_isthmuscingulate	0.463	0.487
			rh_lateraloccipital	0.065	0.347
			rh_lateralorbitofrontal	0.309	0.478
			rh_lingual	0.031	0.283
			rh_medialorbitofrontal	0.487	0.487
			rh_middletemporal	0.141	0.369
			rh_paracentral	0.346	0.478
			rh_parahippocampal	0.097	0.365
			rh_parsopercularis	0.397	0.478
			rh_parsorbitalis	0.075	0.347
			rh_parstriangularis	0.374	0.478
			rh_pericalcarine	0.038	0.283
			rh_postcentral	0.315	0.478
			rh_posteriorcingulate	0.469	0.487
			rh_precentral	0.295	0.478
			rh_precuneus	0.141	0.369
			rh_rostralanteriorcingulate	0.169	0.383
			rh_rostralmiddlefrontal	0.401	0.478
			rh_superiorfrontal	0.389	0.478
			rh_superiorparietal	0.086	0.358
			rh_superiortemporal	0.428	0.485
			rh_supramarginal	0.389	0.478
			rh_temporalpole	0.115	0.369
			rh_transversetemporal	0.304	0.478
		late preHD	lh_bankssts	0.343	0.503
			lh_caudalanteriorcingulate	0.132	0.494
			lh_caudalmiddlefrontal	0.290	0.494
			lh_cuneus	0.438	0.494
			lh_entorhinal	0.331	0.494
			lh_frontalpole	0.217	0.494
			lh_fusiform	0.477	0.494
			lh_inferiorparietal	0.464	0.494
			lh_inferiortemporal	0.461	0.494
			lh_insula	0.195	0.494
			lh_isthmuscingulate	0.384	0.494
			lh_lateraloccipital	0.447	0.494
			lh_lateralorbitofrontal	0.190	0.494
			lh_lingual	0.524	0.494
			lh_medialorbitofrontal	0.167	0.494
			lh_middletemporal	0.212	0.494

			lh_paracentral	0.169	0.494
			lh_parahippocampal	0.235	0.494
			lh_parsopercularis	0.287	0.494
			lh_parsorbitalis	0.423	0.494
			lh_parstriangularis	0.486	0.494
			lh_pericalcarine	0.484	0.494
			lh_postcentral	0.076	0.494
			lh_posteriorcingulate	0.068	0.494
			lh_precentral	0.086	0.494
			lh_precuneus	0.160	0.494
			lh_rostralanteriorcingulate	0.078	0.494
			lh_rostralmiddlefrontal	0.140	0.494
			lh_superiorfrontal	0.153	0.494
			lh_superiorparietal	0.442	0.494
			lh_superiortemporal	0.151	0.493
			lh_supramarginal	0.135	0.493
			lh_temporalpole	0.465	0.491
			lh_transversetemporal	0.144	0.491
			rh_bankssts	0.279	0.491
			rh_caudalanteriorcingulate	0.138	0.491
			rh_caudalmiddlefrontal	0.448	0.491
			rh_cuneus	0.436	0.491
			rh_entorhinal	0.219	0.485
			rh_frontalpole	0.310	0.485
			rh_fusiform	0.469	0.485
			rh_inferiorparietal	0.268	0.485
			rh_inferiortemporal	0.344	0.485
			rh_insula	0.124	0.485
			rh_isthmuscingulate	0.298	0.485
			rh_lateraloccipital	0.435	0.485
			rh_lateralorbitofrontal	0.366	0.485
			rh_lingual	0.474	0.485
			rh_medialorbitofrontal	0.122	0.485
			rh_middletemporal	0.251	0.485
			rh_paracentral	0.101	0.485
			rh_parahippocampal	0.284	0.485
			rh_parsopercularis	0.236	0.485
			rh_parsorbitalis	0.384	0.485
			rh_parstriangularis	0.366	0.485
			rh_pericalcarine	0.360	0.485

			rh_postcentral	0.071	0.485
			rh_posteriorcingulate	0.108	0.485
			rh_precentral	0.096	0.485
			rh_precuneus	0.294	0.485
			rh_rostralanteriorcingulate	0.042	0.485
			rh_rostralmiddlefrontal	0.190	0.485
			rh_superiorfrontal	0.108	0.485
			rh_superiorparietal	0.394	0.485
			rh_superiortemporal	0.188	0.485
			rh_supramarginal	0.233	0.485
			rh_temporalpole	0.425	0.485
			rh_transversetemporal	0.082	0.485
		mHD	lh_bankssts	0.096	0.204
			lh_caudalanteriorcingulate	0.050	0.186
			lh_caudalmiddlefrontal	0.058	0.186
			lh_cuneus	0.350	0.371
			lh_entorhinal	0.005	0.153
			lh_frontalpole	0.263	0.323
			lh_fusiform	0.041	0.186
			lh_inferiorparietal	0.097	0.204
			lh_inferiortemporal	0.028	0.186
			lh_insula	0.222	0.279
			lh_isthmuscingulate	0.162	0.224
			lh_lateraloccipital	0.065	0.186
			lh_lateralorbitofrontal	0.033	0.186
			lh_lingual	0.421	0.429
			lh_medialorbitofrontal	0.124	0.206
			lh_middletemporal	0.082	0.199
			lh_paracentral	0.117	0.204
			lh_parahippocampal	0.138	0.206
			lh_parsopercularis	0.133	0.206
			lh_parsorbitalis	0.186	0.247
			lh_parstriangularis	0.108	0.204
			lh_pericalcarine	0.303	0.337
			lh_postcentral	0.069	0.186
			lh_posteriorcingulate	0.009	0.162
			lh_precentral	0.055	0.186
			lh_precuneus	0.279	0.323
			lh_rostralanteriorcingulate	0.274	0.323
			lh_rostralmiddlefrontal	0.012	0.162

			lh_superiorfrontal	0.031	0.186
			lh_superiorparietal	0.135	0.206
			lh_superiortemporal	0.073	0.186
			lh_supramarginal	0.052	0.186
			lh_temporalpole	0.210	0.275
			lh_transversetemporal	0.107	0.204
			rh_bankssts	0.114	0.204
			rh_caudalanteriorcingulate	0.060	0.186
			rh_caudalmiddlefrontal	0.094	0.204
			rh_cuneus	0.423	0.429
			rh_entorhinal	0.015	0.162
			rh_frontalpole	0.296	0.335
			rh_fusiform	0.064	0.186
			rh_inferiorparietal	0.069	0.186
			rh_inferiortemporal	0.018	0.162
			rh_insula	0.107	0.204
			rh_isthmuscingulate	0.170	0.232
			rh_lateraloccipital	0.221	0.279
			rh_lateralorbitofrontal	0.011	0.162
			rh_lingual	0.447	0.447
			rh_medialorbitofrontal	0.408	0.426
			rh_middletemporal	0.146	0.211
			rh_paracentral	0.116	0.204
			rh_parahippocampal	0.140	0.206
			rh_parsopercularis	0.106	0.204
			rh_parsorbitalis	0.274	0.323
			rh_parstriangularis	0.038	0.186
			rh_pericalcarine	0.280	0.323
			rh_postcentral	0.126	0.206
			rh_posteriorcingulate	0.019	0.162
			rh_precentral	0.087	0.204
			rh_precuneus	0.140	0.206
			rh_rostralanteriorcingulate	0.341	0.371
			rh_rostralmiddlefrontal	0.004	0.153
			rh_superiorfrontal	0.074	0.186
			rh_superiorparietal	0.062	0.186
			rh_superiortemporal	0.064	0.186
			rh_supramarginal	0.066	0.186
			rh_temporalpole	0.344	0.371
			rh_transversetemporal	0.160	0.224

structural connectivity	early preHD	lh_bankssts	0.312	0.424
		lh_caudalanteriorcingulate	0.033	0.148
		lh_caudalmiddlefrontal	0.406	0.476
		lh_cuneus	0.027	0.148
		lh_entorhinal	0.371	0.464
		lh_frontalpole	0.167	0.315
		lh_fusiform	0.010	0.140
		lh_inferiorparietal	0.341	0.455
		lh_inferiortemporal	0.187	0.332
		lh_insula	0.004	0.140
		lh_isthmuscingulate	0.388	0.471
		lh_lateraloccipital	0.032	0.148
		lh_lateralorbitofrontal	0.196	0.332
		lh_lingual	0.211	0.341
		lh_medialorbitofrontal	0.286	0.397
		lh_middletemporal	0.133	0.299
		lh_paracentral	0.031	0.148
		lh_parahippocampal	0.091	0.258
		lh_parsopercularis	0.602	0.602
		lh_parsorbitalis	0.558	0.566
		lh_parstriangularis	0.554	0.566
		lh_pericalcarine	0.023	0.148
		lh_postcentral	0.167	0.315
		lh_posteriorcingulate	0.071	0.218
		lh_precentral	0.255	0.385
		lh_precuneus	0.372	0.464
		lh_rostralanteriorcingulate	0.267	0.386
		lh_rostralmiddlefrontal	0.082	0.244
		lh_superiorfrontal	0.007	0.140
		lh_superiorparietal	0.040	0.148
		lh_superiortemporal	0.204	0.338
		lh_supramarginal	0.132	0.299
		lh_temporalpole	0.492	0.540
		lh_transversetemporal	0.438	0.504
		rh_bankssts	0.166	0.315
		rh_caudalanteriorcingulate	0.016	0.140
		rh_caudalmiddlefrontal	0.473	0.536
		rh_cuneus	0.048	0.154
		rh_entorhinal	0.376	0.464
		rh_frontalpole	0.194	0.332

			rh_fusiform	0.037	0.148
			rh_inferiorparietal	0.137	0.299
			rh_inferiortemporal	0.031	0.148
			rh_insula	0.044	0.148
			rh_isthmuscingulate	0.370	0.464
			rh_lateraloccipital	0.014	0.140
			rh_lateralorbitofrontal	0.510	0.550
			rh_lingual	0.044	0.148
			rh_medialorbitofrontal	0.110	0.298
			rh_middletemporal	0.015	0.140
			rh_paracentral	0.017	0.140
			rh_parahippocampal	0.153	0.315
			rh_parsopercularis	0.261	0.385
			rh_parsorbitalis	0.114	0.298
			rh_parstriangularis	0.171	0.315
			rh_pericalcarine	0.027	0.148
			rh_postcentral	0.257	0.385
			rh_posteriorcingulate	0.043	0.148
			rh_precentral	0.493	0.540
			rh_precuneus	0.283	0.397
			rh_rostralanteriorcingulate	0.121	0.299
			rh_rostralmiddlefrontal	0.156	0.315
			rh_superiorfrontal	0.008	0.140
			rh_superiorparietal	0.230	0.364
			rh_superiortemporal	0.552	0.566
			rh_supramarginal	0.123	0.299
			rh_temporalpole	0.536	0.566
			rh_transversetemporal	0.402	0.476
		late preHD	lh_bankssts	0.500	0.470
			lh_caudalanteriorcingulate	0.100	0.392
			lh_caudalmiddlefrontal	0.150	0.470
			lh_cuneus	0.500	0.470
			lh_entorhinal	0.150	0.392
			lh_frontalpole	0.400	0.470
			lh_fusiform	0.150	0.392
			lh_inferiorparietal	0.400	0.470
			lh_inferiortemporal	0.000	0.340
			lh_insula	0.350	0.470
			lh_isthmuscingulate	0.700	0.470
			lh_lateraloccipital	0.150	0.392
			lh_lateralorbitofrontal	0.400	0.470

			lh_lingual	0.300	0.426
			lh_medialorbitofrontal	0.350	0.470
			lh_middletemporal	0.100	0.426
			lh_paracentral	0.000	0.023
			lh_parahippocampal	0.400	0.474
			lh_parsopercularis	0.150	0.435
			lh_parsorbitalis	0.300	0.470
			lh_parstriangularis	0.550	0.470
			lh_pericalcarine	0.550	0.470
			lh_postcentral	0.050	0.068
			lh_posteriorcingulate	0.000	0.023
			lh_precentral	0.000	0.286
			lh_precuneus	0.000	0.286
			lh_rostralanteriorcingulate	0.200	0.409
			lh_rostralmiddlefrontal	0.550	0.470
			lh_superiorfrontal	0.050	0.392
			lh_superiorparietal	0.200	0.470
			lh_superiortemporal	0.450	0.470
			lh_supramarginal	0.050	0.392
			lh_temporalpole	0.450	0.470
			lh_transversetemporal	0.300	0.470
			rh_bankssts	0.450	0.470
			rh_caudalanteriorcingulate	0.100	0.409
			rh_caudalmiddlefrontal	0.300	0.470
			rh_cuneus	0.100	0.470
			rh_entorhinal	0.100	0.426
			rh_frontalpole	0.450	0.486
			rh_fusiform	0.050	0.286
			rh_inferiorparietal	0.300	0.470
			rh_inferiortemporal	0.150	0.426
			rh_insula	0.400	0.470
			rh_isthmuscingulate	0.350	0.470
			rh_lateraloccipital	0.150	0.392
			rh_lateralorbitofrontal	0.500	0.535
			rh_lingual	0.100	0.422
			rh_medialorbitofrontal	0.000	0.392
			rh_middletemporal	0.300	0.392
			rh_paracentral	0.000	0.043
			rh_parahippocampal	0.300	0.470
			rh_parsopercularis	0.150	0.392

			rh_parsorbitalis	0.450	0.470
			rh_parstriangularis	0.500	0.470
			rh_pericalcarine	0.350	0.470
			rh_postcentral	0.000	0.108
			rh_posteriorcingulate	0.000	0.023
			rh_precentral	0.100	0.286
			rh_precuneus	0.100	0.392
			rh_rostralanteriorcingulate	0.050	0.392
			rh_rostralmiddlefrontal	0.300	0.458
			rh_superiorfrontal	0.000	0.422
			rh_superiorparietal	0.300	0.470
			rh_superiortemporal	0.400	0.470
			rh_supramarginal	0.350	0.426
			rh_temporalpole	0.100	0.392
			rh_transversetemporal	0.100	0.392
		mHD	lh_bankssts	0.198	0.453
			lh_caudalanteriorcingulate	0.381	0.453
			lh_caudalmiddlefrontal	0.107	0.429
			lh_cuneus	0.247	0.453
			lh_entorhinal	0.466	0.480
			lh_frontalpole	0.363	0.453
			lh_fusiform	0.331	0.453
			lh_inferiorparietal	0.017	0.276
			lh_inferiortemporal	0.337	0.453
			lh_insula	0.360	0.453
			lh_isthmuscingulate	0.468	0.480
			lh_lateraloccipital	0.446	0.480
			lh_lateralorbitofrontal	0.361	0.453
			lh_lingual	0.372	0.453
			lh_medialorbitofrontal	0.286	0.453
			lh_middletemporal	0.064	0.395
			lh_paracentral	0.234	0.453
			lh_parahippocampal	0.266	0.453
			lh_parsopercularis	0.031	0.276
			lh_parsorbitalis	0.320	0.453
			lh_parstriangularis	0.203	0.453
			lh_pericalcarine	0.414	0.470
			lh_postcentral	0.003	0.276
			lh_posteriorcingulate	0.090	0.395
			lh_precentral	0.014	0.276

			lh_precuneus	0.447	0.480
			lh_rostralanteriorcingulate	0.260	0.453
			lh_rostralmiddlefrontal	0.090	0.429
			lh_superiorfrontal	0.126	0.442
			lh_superiorparietal	0.157	0.453
			lh_superiortemporal	0.035	0.276
			lh_supramarginal	0.043	0.333
			lh_temporalpole	0.232	0.453
			lh_transversetemporal	0.039	0.333
			rh_bankssts	0.244	0.453
			rh_caudalanteriorcingulate	0.350	0.453
			rh_caudalmiddlefrontal	0.156	0.452
			rh_cuneus	0.369	0.453
			rh_entorhinal	0.341	0.453
			rh_frontalpole	0.402	0.453
			rh_fusiform	0.464	0.480
			rh_inferiorparietal	0.024	0.276
			rh_inferiortemporal	0.111	0.429
			rh_insula	0.430	0.480
			rh_isthmuscingulate	0.436	0.491
			rh_lateraloccipital	0.253	0.453
			rh_lateralorbitofrontal	0.463	0.480
			rh_lingual	0.328	0.453
			rh_medialorbitofrontal	0.347	0.453
			rh_middletemporal	0.207	0.453
			rh_paracentral	0.277	0.453
			rh_parahippocampal	0.450	0.480
			rh_parsopercularis	0.180	0.453
			rh_parsorbitalis	0.229	0.453
			rh_parstriangularis	0.323	0.453
			rh_pericalcarine	0.262	0.453
			rh_postcentral	0.014	0.276
			rh_posteriorcingulate	0.107	0.429
			rh_precentral	0.170	0.453
			rh_precuneus	0.224	0.453
			rh_rostralanteriorcingulate	0.294	0.453
			rh_rostralmiddlefrontal	0.110	0.429
			rh_superiorfrontal	0.309	0.453
			rh_superiorparietal	0.070	0.395
			rh_superiortemporal	0.037	0.276

			rh_supramarginal	0.109	0.429
			rh_temporalpole	0.456	0.491
			rh_transversetemporal	0.177	0.453
subcortex	functional connectivity	early preHD	Laccumb	0.034	0.181
			Lamyg	0.232	0.248
			Lcaud	0.143	0.181
			Lhippo	0.120	0.181
			Lpal	0.104	0.181
			Lput	0.083	0.181
			Lthal	0.059	0.181
			Raccumb	0.046	0.181
			Ramyg	0.248	0.248
			Rcaud	0.163	0.190
			Rhippo	0.143	0.181
			Rpal	0.116	0.181
			Rput	0.084	0.181
			Rthal	0.071	0.181
		late preHD	Laccumb	0.034	0.180
			Lamyg	0.253	0.266
			Lcaud	0.159	0.186
			Lhippo	0.128	0.186
			Lpal	0.090	0.180
			Lput	0.075	0.180
			Lthal	0.051	0.180
			Raccumb	0.062	0.180
			Ramyg	0.266	0.266
			Rcaud	0.155	0.186
			Rhippo	0.134	0.186
			Rpal	0.117	0.186
			Rput	0.088	0.180
			Rthal	0.070	0.180
		mHD	Laccumb	0.030	0.188
			Lamyg	0.231	0.249
			Lcaud	0.154	0.196
			Lhippo	0.135	0.188
			Lpal	0.114	0.188
			Lput	0.079	0.188
			Lthal	0.058	0.188
			Raccumb	0.059	0.188
			Ramyg	0.265	0.265

			Rcaud	0.168	0.196
			Rhippo	0.131	0.188
			Rpal	0.118	0.188
			Rput	0.070	0.188
			Rthal	0.080	0.188
	structural connectivity	early preHD	Laccumb	0.119	0.207
			Lamyg	0.211	0.246
			Lcaud	0.209	0.246
			Lhippo	0.404	0.404
			Lpal	0.018	0.207
			Lput	0.081	0.207
			Lthal	0.131	0.207
			Raccumb	0.318	0.342
			Ramyg	0.148	0.207
			Rcaud	0.136	0.207
			Rhippo	0.066	0.207
			Rpal	0.098	0.207
			Rput	0.034	0.207
			Rthal	0.085	0.207
		late preHD	Laccumb	0.109	0.414
			Lamyg	0.221	0.353
			Lcaud	0.203	0.257
			Lhippo	0.414	0.257
			Lpal	0.024	0.210
			Lput	0.078	0.210
			Lthal	0.122	0.210
			Raccumb	0.328	0.210
			Ramyg	0.150	0.210
			Rcaud	0.144	0.210
			Rhippo	0.079	0.210
			Rpal	0.109	0.210
			Rput	0.035	0.210
			Rthal	0.071	0.210
		mHD	Laccumb	0.109	0.205
			Lamyg	0.221	0.257
			Lcaud	0.212	0.257
			Lhippo	0.403	0.403
			Lpal	0.025	0.205
			Lput	0.088	0.205

			Lthal	0.132	0.205
			Raccumb	0.309	0.333
			Ramyg	0.135	0.205
			Rcaud	0.147	0.205
			Rhippo	0.076	0.205
			Rpal	0.093	0.205
			Rput	0.039	0.205
			Rthal	0.090	0.205

Table S22 P values in the epicenter analysis

Non-parametric spin permutation test, two-tailed, FDR correction at a P<0.05, DF = 66FDR
FDR, false-discovery rate; mHD, manifest HD; preHD, premanifest HD.

Table S23 P values in the organizational principles analysis

		Population	Pspin	FDR-corrected Pspin
STRUCTURAL	Laminar similarity	early preHD	0.555	0.833
		late preHD	0.086	0.128
		mHD	0.956	0.956
	White matter connectivity	Early preHD	0.162	0.243
		Late preHD	0.032	0.096
		mHD	0.580	0.580
	Euclidean distance	Early preHD	0.233	0.349
		Late preHD	0.017	0.051
		mHD	0.966	0.966
FUNCTIONAL	FDG-PET	early preHD	0.812	0.812
		late preHD	0.011	0.016
		mHD	0.007	0.016
	rsfMRI	early preHD	0.389	0.389
		late preHD	0.138	0.237
		mHD	0.158	0.237
	dynamic rsfMRI	early preHD	0.419	0.419
		late preHD	0.097	0.222
		mHD	0.148	0.222
	MEG	early preHD	0.078	0.117
		late preHD	0.024	0.072
		mHD	0.757	0.757
CELL AUTONOMOUS	Gene coexpression	early preHD	0.128	0.298
		late preHD	0.021	0.031
		mHD	0.014	0.031
	Receptor similarity	early preHD	0.016	0.048
		late preHD	0.110	0.128
		mHD	0.128	0.221

mHD, manifest Huntington's disease; preHD, premanifest Huntington's disease.
Statistical significance was assessed through non-parametric FDR-corrected spin permutation tests, two-tailed, DF = 66
FDR, false-discovery rate; mHD, manifest HD; preHD, premanifest HD.

Table S24 P values in the receptome analysis

Gradient	Population	Pspin	FDR-corrected Pspin
Gradient 1	Early preHD	0.001	0.003
	Late preHD	0.021	0.0315
	mHD	0.911	0.911
Gradient 2	Early preHD	0.898	0.898
	Late preHD	0.441	0.898
	mHD	0.711	0.898
Gradient 3	Early preHD	0.286	0.858
	Late preHD	0.725	0.868
	mHD	0.868	0.868

Statistical significance was assessed through FDR-corrected spin permutation tests, two-tailed, DF = 98
FDR, false-discovery rate; mHD, manifest Huntington's disease; preHD, premanifest Huntington's disease

Table S25 P values PET neurotransmitter analysis

	Pspin	FDR-corrected Pspin
Early preHD	0.012	0.019
Late preHD	0.019	0.019
mHD	0.013	0.019

FDR-corrected spin permutation tests, one-sided, N = 68 regions

FDR, false-discovery rate; mHD, manifest Huntington's disease; preHD, premanifest Huntington's disease

Table S26 P values in the autoradiography neurotransmitter analysis

		Pspin	FDR-corrected Pspin
SUPRAGRANULAR	Early preHD	0.006	0.101
	Late preHD	0.101	0.101
	mHD	0.067	0.019
GRANULAR	Early preHD	0.003	0.010
	Late preHD	0.034	0.051
	mHD	0.359	0.359
INFRAGRANULAR	Early preHD	0.239	0.359
	Late preHD	0.028	0.085
	mHD	0.664	0.664

FDR-corrected spin permutation tests, one-sided, N = 68 regions

False-discovery rate; mHD, manifest Huntington's disease; preHD, premanifest Huntington's disease.

Supplementary note

The distribution of $GABA_A$ receptors in granular and infragranular layers was also associated with hyperconnectivity in early preHD participants. GABAergic receptors have been found to be upregulated in the frontal cortex of HD[1], as a potential compensatory response to the excitotoxic cascade initiated before the onset of cell loss, a finding recently confirmed in an ovine model of HD[2].

We found an association between the densities of vesicular acetylcholine transporter, a surrogate marker for the density of cholinergic nerve terminals[3,4], and connectivity changes in early and late preHD, but not with the distribution of M_1 muscarinic cholinergic receptors. The cortical input to the striatum arises from pyramidal tract and intratelencephalic neurons[5]. The activation nicotinic acetylcholine receptors in cholinergic interneurons connecting to pyramidal tract neurons results in the release of glutamate and eventual corticostriatal signalling[5], a mechanism involved in excitotoxicity in HD[6]. A recent study suggested that muscarinic receptors are also involved in HD pathogenesis. However this work was performed in the zQ175 mouse model[6] at a time point where there are severe motor symptoms and extensive neuronal loss[7] while we focused on human participants with none or mild symptoms and mild neurodegenerative changes. Our results support that nicotinic, rather than muscarinic, receptors are involved in the pathogenesis of HD from very early preHD stages.

Other associations were less robust. Dopaminergic D_1 and D_2 receptors were moderately associated with connectivity changes in mHD, consistent with the presence of movement disorders in this cohort, while the dopamine transporter distribution was associated with connectivity in early and late preHD. Dopaminergic receptors and transporters interact[8] and it is tempting to speculate that our analysis point to alterations in the dopamine transporter resulting in eventual neuronal loss of dopaminergic regions. Histaminergic H_3 receptors, also associated with connectivity changes in mHD in our study, form heterodimers with D_1 receptors, protecting dopaminergic neurons from degeneration, a mechanism considered as a therapeutic target in HD[9].

References

1 Lloyd KG, Dreksler S, Bird ED. Alterations in 3H-GABA binding in Huntington's chorea. *Life Sci* 1977; **21**: 747–53.

2 Jacobsen JC, Patassini S, Rudiger SR, *et al.* Evidence for glutamate excitotoxicity that occurs before the onset of cell loss and motor symptoms in an ovine Huntington's Disease model. 2023.

3 Aghourian M, Soucy J, Gauthier S, Kostikov A, Gravel P, Bedard M. Quanti fi cation of brain cholinergic denervation in Alzheimer ' s disease using PET imaging with [18 F] -FEOBV. 2017; : 1531–8.

4 Bedard M, Aghourian M, Legault-denis C, Montplaisir J. Brain cholinergic alterations in idiopathic REM sleep behaviour disorder : a PET imaging study with 18 F-FEOBV. 2019; **58**: 35–41.

5 Morgenstern NA, Isidro AF, Israely I, Costa RM. Pyramidal tract neurons drive amplification of excitatory inputs to striatum through cholinergic interneurons. 2022; **4315**: 1–15.

6 Pancani T, Day M, Tkatch T, *et al.* Cholinergic deficits selectively boost cortical intratelencephalic control of striatum in male Huntington's disease model mice. *Nat Commun* 2023; **14**: 1398.

7 Heikkinen T, Lehtimäki K, Vartiainen N, *et al.* Characterization of Neurophysiological and Behavioral Changes, MRI Brain Volumetry and 1H MRS in zQ175 Knock-In Mouse Model of Huntington's Disease. *PLoS One* 2012; **7**. DOI:10.1371/journal.pone.0050717.

8 Lee FJS, Pei L, Vukusic B, Fletcher PJ, Liu F. Dopamine transporter cell surface localization facilitated by a direct interaction with the dopamine D2 receptor. 2007; **26**: 2127–36.

9 Moreno-Delgado D, Puigdellívol M, Moreno E, *et al.* Modulation of dopamine D1 receptors via histamine H3 receptors is a novel therapeutic target for Huntington's disease. *Elife* 2020; **9**: 1–31.
